# Exon-Enriched Libraries Reveal Large Genic Differences Between *Aedes aegypti* from Senegal, West Africa, and Populations Outside Africa

**DOI:** 10.1534/g3.116.036053

**Published:** 2016-12-19

**Authors:** Laura B. Dickson, Corey L. Campbell, Punita Juneja, Francis M. Jiggins, Massamba Sylla, William C. Black

**Affiliations:** * Department of Microbiology, Immunology and Pathology, Colorado State University, Fort Collins, Colorado 80523; † Department of Genetics, University of Cambridge, CB24 6BG, United Kingdom

**Keywords:** *Aedes aegypti*, West Africa, genome, population genomics, subspecies

## Abstract

*Aedes aegypti* is one of the most studied mosquito species, and the principal vector of several arboviruses pathogenic to humans. Recently failure to oviposit, low fecundity, and poor egg-to-adult survival were observed when *Ae. aegypti* from Senegal (*SenAae*) West Africa were crossed with *Ae. aegypti* (*Aaa*) from outside of Africa, and in *SenAae* intercrosses. Fluorescent *in situ* hybridization analyses indicated rearrangements on chromosome 1, and pericentric inversions on chromosomes 2 and 3. Herein, high throughput sequencing (HTS) of exon-enriched libraries was used to compare chromosome-wide genetic diversity among *Aaa* collections from rural Thailand and Mexico, a sylvatic collection from southeastern Senegal (PK10), and an urban collection from western Senegal (Kaolack). Sex-specific polymorphisms were analyzed in Thailand and PK10 to assess genetic differences between sexes. Expected heterozygosity was greatest in *SenAae*. F_ST_ distributions of 15,735 genes among all six pairwise comparisons of the four collections indicated that Mexican and Thailand collections are genetically similar, while F_ST_ distributions between PK10 and Kaolack were distinct. All four comparisons of *SenAae* with *Aaa* indicated extreme differentiation. F_ST_ was uniform between sexes across all chromosomes in Thailand, but were different, especially on the sex autosome 1, in PK10. These patterns correlate with the reproductive isolation noted earlier. We hypothesize that cryptic *Ae. aegypti* taxa may exist in West Africa, and the large genic differences between *Aaa* and *SenAae* detected in the present study have accumulated over a long period following the evolution of chromosome rearrangements in allopatric populations that subsequently cause reproductive isolation when these populations became sympatric.

The mosquito, *Aedes aegypti* (L), is the principal vector in tropical and subtropical regions world-wide of dengue (DENV1–4)(Guzman and Harris 2015), Yellow Fever (YF) (Beasley *et al.* 2015) and Zika (Li *et al.* 2012) flaviviruses, and of the Chikungunya alphavirus (Lo Presti *et al.* 2016). The ecology and population biology of the species is well understood (Mattingly 1957), and the ease of colonization and laboratory manipulation have helped it to become the most studied of all mosquito species. There are two recognized subspecies of *Ae. aegypti*: *Ae. aegypti* (*Aaa*) is a domestic form that has spread circumglobally between 35° north and south latitudes, typically in close association with humans, while *Ae. aegypti formosus (Aaf)* is a sylvan mosquito that occurs primarily in forest habitats. Most studies of these subspecies have occurred in East Africa, where the subspecies are sympatric. McClelland (1974) formally defined *Aaf* populations as having a dark black cuticle, no pale scales on the first abdominal tergite, breeding in natural containers such as tree holes, and feeding primarily on wild animals. In contrast, *Aaa* is a more domestic subspecies that has spread out of Africa. *Aaa* breeds in artificial containers provided by humans, will breed indoors, and has a preference for feeding on human blood. It is described as being a lighter colored mosquito, with a range of pale scaling on the abdominal tergites. Presumably, *Aaa* arose from ancestral *Aaf*, and eventually became adapted to human habitats. It then spread from Africa to many different tropical and subtropical regions of the world through human commerce and exploration (Tabachnick 1991).

Detailed mark-release-recapture studies in Kenya (Trpis and Hausermann 1975) demonstrated that immature mosquitoes collected from sylvan, peridomestic, or domestic breeding containers showed an overwhelming preference for their respective habitat as adults. Laboratory experiments crossing *Aaa* and *Aaf* from Kenya showed no evidence of assortative mating between the two subspecies (Moore 1979), and found no decrease in fecundity in hybrids, nor any morphological defects. In rural Kenya, *Aaf* collected from the forest had very different allozyme frequencies when compared to *Aaa* collected indoors, although some populations used in the study were laboratory colonies several generations from the field (Scott and McClelland 1975). Another study in Kenya showed that *Aaa* populations within villages are panmictic, but genetically differentiated from *Aaa* in other villages, even those <2 km away. This suggested that gene flow between villages is infrequent, and *Aaf* living in the intervening forest do not serve as a “genetic bridge,” so that *Aaf* and *Aaa* represent separate breeding populations (Tabachnick and Powell 1978). Further investigation demonstrated that *Aaf* collected from the forest or disturbed areas around villages (*e.g.*, coconut groves) were genetically distinct from *Aaa* collections made indoors (Tabachnick *et al.* 1979).

In contrast, *Ae. aegypti* in West Africa has received relatively little attention. While scale patterns correlate with genetic differences in East Africa (Tabachnick *et al.* 1979), this is not the case in West Africa (Huber *et al.* 2008; Moore *et al.* 2013; Paupy *et al.* 2008, 2010; Sylla *et al.* 2009), where cuticle color is predominantly black, with a continuum of scaling patterns. Collections from West Africa with varied scale patterns have been sampled in both the dry and rainy seasons from different vegetation zones, and from domestic *vs.* sylvan habitats. Regardless of collection site, most variation was associated with geographic distance, vegetative zones, ecological habitats, and season, rather than marker type or scale pattern (Brown *et al.* 2014, 2011; Huber *et al.* 2008; Paupy *et al.* 2008; Sylla *et al.* 2009). Accordingly, we designated *Ae. aegypti* in West Africa as *SenAae* because cuticle color is predominantly black (therefore not *Aaa*), and mosquitoes collected in both sylvatic and urban sites exhibit a continuum of scaling patterns (therefore not *Aaf*).

In an attempt to clarify genetic relationships among, and within, West African *Ae. aegypti*, we recently reported crossing experiments of *Aaa* P_1_ laboratory strains with Senegal *Ae. aegypti* (*SenAae*) (Dickson *et al.* 2016), wherein we were unable to build intercross families due to poor F_1_ oviposition and low F_2_ egg-to-adult survival. Egg hatch was predicted based upon Haldane’s rule (Haldane 1922) to be low when a female mates with a hybrid F_1_ male, but average when a male mates with a hybrid F_1_ female. Instead, most females mated to hybrid males laid no eggs, and egg-pupal survival was reduced when males mated with hybrid females. Linkage and fluorescent *in situ* hybridization (FISH) analyses identified a likely rearrangement on chromosome 1, and identified two rDNA cistrons on that chromosome, and pericentric inversions on chromosomes 2 and 3 (Dickson *et al.* 2016). The latter results suggest that the reproductive isolation observed within *SenAae* may be associated with chromosome rearrangements. These observations differ markedly from the 1979 study of hybridization and mating behavior between *Aaa* and *Aaf* in East Africa, which found no evidence of hybrid breakdown or of assortative mating (Moore 1979). The goal of the current study was to use high throughput sequencing (HTS) of exon-enriched gDNA libraries to assess the degree of genetic differentiation between two interfertile *SenAae* (called PK10 and Kaolack), and two interfertile *Aaa* (Thailand and Mexico) collections. Dual replicate pools of equal numbers of males and females were used for these comparisons. Expected heterozygosity (H_exp_) in each collection was compared to give an indication of the relative levels of within-collection genetic diversity. These analyses were extended by assessing sex-specific polymorphisms for the Thailand (*Aaa*) and PK10 (*SenAae*) collections. Standardized variance in SNP frequencies (F_ST_) was compared within and between *Aaa* and *SenAae*, and between females and males in the Thailand (*Aaa*) and PK10 (*SenAae*) collections. SNP frequencies were significantly different among sexes in PK10, with the largest differences occurring on sex chromosome 1, but were homogeneous across all chromosomes in the Thailand collection.

## Materials and Methods

### Mosquito collections

Senegalese *Ae. aegypti* populations were collected as larvae near domestic sites in urban and village environments, as well as from sylvatic habitats throughout Senegal (Sylla *et al.* 2009) (Supplemental Material, Figure S1). In February 2010, *Ae. aegypti* were collected in Kaolack (14°9′22.35′′N, 16°4′32.31′′W) in West Central Senegal, and in the PK10 forest (12°36′43.00′′N, 12°14′46.80′′W) in southeastern Senegal in September 2011. A complete description of mosquitoes found in PK10 has been published (Sylla *et al.* 2013). The PK10 colony was established from larvae taken from treeholes at the forest–savannah margin (Sylla *et al.* 2013). Both 2009 and 2011 PK10 collections consisted of adults without scales on the first abdominal tergite (McClelland 1974). The Merida, Mexico (Vergel) (20°57′16.82′′N, 89°35′20.24′′W) collection of *Aaa* was collected in 2007. The Pai Lom, Mae Sot Thailand (16°42′46.54′′N, 98°34′28.74′′E) 2002 collection of *Aaa* consisted of DNA individually isolated from adults (Bosio *et al.* 2005). Sex-specific polymorphisms were analyzed in PK10 (*SenAae*) and Thailand (*Aaa*), but not in Mexico (*Aaa*) or Kaolack (*SenAae*) collections because mosquitoes were not identified to sex prior to DNA isolation.

Larvae were removed mostly from artificial containers (*e.g.*, tires, water storage containers, and discarded trash), reared to adults, blood fed on the senior author’s arm, and collected eggs were returned to Fort Collins where they were established as laboratory colonies. In each collection, these were raised to adults, transferred to 1-pint cages, anesthetized with Triethylamine (FlyNap Carolina Biological Supply Company, Burlington, NC), and classified according to the presence/absence of scales on the first abdominal tergite (McClelland 1974; Sylla *et al.* 2009, 2013). At least 50 adults of each sex were individually identified and stored in Purell Advanced Hand Sanitizer for eventual extraction of DNA (Black and DuTeau 1997). All colonized adults were maintained at 28°, 70–80% relative humidity, and for a 12:12 hr photoperiod.

### Sequencing

HTS of exon-enriched libraries (Juneja *et al.* 2015) was used to quantify the extent of genomic differences within, and among, the four *Ae. aegypti* collections analyzed in this study, and between sexes within PK10 (*SenAae*) and Thailand (*Aaa*) collections. Twelve libraries were constructed: (1) PK10 males replicate 1 (*n* =12 mosquitoes), (2) PK10 males replicate 2 (*n* = 12), (3) PK10 females replicate 1 (*n* = 12), (4) PK10 females replicate 2 (*n* = 12), (5) Thailand males replicate 1 (*n* = 12), (6) Thailand males replicate 2 (*n* = 12), (7) Thailand females replicate 1, (8) Thailand females replicate 2 (*n* = 12), (9) Kaolack equal numbers of both sexes replicate 1 (*n* = 14), (10) Kaolack equal numbers of both sexes replicate 2 (*n* = 14), (11) Merida, Mexico equal numbers of both sexes replicate 1 (*n* = 22), and (12) Merida, Mexico equal numbers of both sexes replicate 2 (*n* = 22). Thus, each of the four collections was represented by 28, 44, or 48 individuals.

Prior to pooling, DNA in individual mosquitoes was quantified using Pico Green (Life Technologies, Thermo Fisher Scientific Inc.), and equal amounts of DNA per mosquito were pooled. A Covaris S2 sonicator (Covaris Ltd, Brighton, U.K.) sheared pooled DNA to an average size of 500 bp. Sonication conditions were: duty cycle 10%, intensity 5.0, cycles per burst 200, duration 40 sec, mode frequency sweeping, displayed power 23 W, and Temperature 5.5°–6°. Each TruSeq DNA LT (v.2) library was prepared using 1 μg of sheared genomic DNA following the manufacturer’s recommendations. Equimolar quantities of prepared libraries were pooled and enriched for coding sequences by exome capture using custom SeqCap EZ Developer probes (Nimblegen) (Juneja *et al.* 2015). Overlapping probes covering the protein coding sequence (not including UTRs) in the AaegL3.3 gene annotations (https://www.vectorbase.org/organisms/aedes-aegypti/liverpool-lvp/AaegL3.3) were produced by Nimblegen based on exonic coordinates specified by the Jiggins laboratory. In total, 26.7 Mb of the genome (2%) was targeted for enrichment. Exome capture coordinates are available at https://www.jiggins.gen.cam.ac.uk/data/Aaegypti_exome.bed. Enrichment followed the Nimblegen SeqCap EZ protocol. Briefly, pooled TruSeq libraries were hybridized to the probes for 64 hr, unbound DNA was washed away, and the targeted DNA was eluted and amplified. These were then sequenced on two lanes of a HiSeq2000 (Illumina) for paired-end 2 × 100 nt sequencing. TruSeq library preparation, exome capture, and sequencing were performed by the High-Throughput Genomics Group at the Wellcome Trust Centre for Human Genetics (Oxford, UK), and produced reads with quality scores >30.

### Alignments and population genetics pipeline

A custom concatenated reference file “All3U” was built based on the Vectorbase AaegL3.3 gene build, and SNPs were called with respect to this build. The reference was used for the detailed analysis of 18,840 transcripts among 15,735 genes. A FORTRAN program used the coordinates of the features of each gene appearing in *Aedes-aegypti* Liverpool_ BASEFEATURES_Aaegl3.3.GTF to extract: (1) 600 bp of the *cis* region 5′ to each annotated transcript (5′ nontranscribed = 5′NTR), (2) all 5′UTRs, (3) exons, (4) introns, (5) 3′UTRs, and (6) a 600 bp *cis* region in the 3′ direction (3′ nontranscribed = 3′NTR). Each SNP was given an ordered SNPID number according to its position in the gene, the location of the gene in the supercontig, and where the supercontig mapped physically (Timoshevskiy *et al.* 2014). There were 1948 mapped genes containing 41,119,004 SNPs on chromosome 1, 3429 genes containing 70,643,307 SNPs on chromosome 2, and 2382 genes with 44,883,168 SNPs on chromosome 3. There were 7862 unmapped genes containing 144,703,188 SNPs. In total there were 15,735 genes containing 301,348,667 SNPs.

Fastq files from each library were aligned to the All3U reference file using GSNAP (v. 2013-10-28), allowing 10% divergence from the reference (Wu and Nacu 2010). SAMtools (Li *et al.* 2009) converted GSNAP outputs to *.mpileup files. The “readcounts” command in Varscan2 (v2.3.5) (Koboldt *et al.* 2012) used the *.mpileup file to report SNPs, with default values of a minimum of 15× coverage in each library, and a minimum base quality of 30 (http://varscan.sourceforge.net/using-varscan.html). A series of FORTRAN programs then read the “readcount” files to produce a flat file containing the SNPID number, the Aaegl3.3 reference nucleotide, its coverage at each sequenced position, and the coverages of insertions or deletions at that position.

The program “Trim” removed all SNPs that had <15 counts, and capped all SNP coverage at 2000 to avoid regions of repetitive DNA. The program “2 × 2” read two files, one from each replicate at a collection site, and produced a single “2 × 2.out” file that contained SNPs that occurred in both replicate datasets for each collection (Mexico, Thailand male, Thailand female, PK10 male, PK10 female, and Kaolack). A program “2 × 2 × 2” then read two 2 × 2.out files to produce a list of SNPs common to a pair of collections. A program “Tables” then read the two “2 × 2.out” files and the list to produce a contingency table for each SNP common to both collections. The contingency file contained: (1) the SNPID coordinate followed by four lines containing (2) the name of the library containing that SNP, and (3) the coverage for each nucleotide at that SNP. To ensure high confidence SNP calls, replicate SNP frequencies were compared using a heterogeneity χ^2^ test with *nid*-1 degrees of freedom, where *nid* is the number of nucleotides and indels segregating at a SNP. Any SNP in which nucleotide frequencies were significantly different between either replicate were discarded. A program read the contingency table file to produce a file that contains for each SNP: (1) the SNPID, (2) the VectorBase Gene ID (*e.g.*, AAEL001234), (3) coverages of all nucleotide/indels in the first collection, (4) coverages of all nucleotide/indels in the second collection, (5) numbers of alternate nucleotides at that SNP, (6) a list of polymorphic nucleotides/indels, (7) the Aaegl3.3 reference nucleotide, (8) the alternate nucleotide (second most common nucleotide, also referred to as the minor allele), (9) mutation type (*i.e.*, “transition,” “transversion,” “insertion,” or “deletion”), (10) whether the mutation is a synonymous or a replacement substitution for SNPs in codons, and (11) whether the SNP resides in a first, second, or third codon position, or occurs within a 5′UTR, 5′NTR, 3′UTR, 3′NTR, or an intron. Transfer RNAs were annotated using the anticodon tool at http://lowelab.ucsc.edu/tRNAscan-SE/ (Schattner *et al.* 2005).

A program was written to calculate for each SNP, a between-collection component $\left(a_{s}\right),$ a within-collection component $\left(b_{s}\right)$, and F_ST_ calculated from $a_{s}$ and $b_{s}\;$following Fumagalli *et al.* (2013), where:

$$a_{s}=\frac{4n_{i}{\left({\hat{p}}_{\left(i,s\right)}-{\hat{p}}_{s}\right)}^{2}+4n_{j}{\left({\hat{p}}_{\left(j,s\right)}-{\hat{p}}_{s}\right)}^{2}-b_{s}}{2\left[2n_{i}n_{j}/\left(n_{i}+n_{j}\right)\right]}$$

and

$$b_{s}=\frac{n_{i}\alpha_{\left(i,s\right)}+n_{j}\alpha_{\left(j,s\right)}}{n_{i}+n_{j}-1}$$

where

$$\alpha_{\left(i,s\right)}=2{\hat{p}}_{\left(i,s\right)}\left(1-{\hat{p}}_{\left(i,s\right)}\right)\;\mathrm{and}\;\alpha_{\left(j,s\right)}=2{\hat{p}}_{\left(j,s\right)}\left(1-{\hat{p}}_{\left(j,s\right)}\right)$$

$\alpha_{\left(i,s\right)}$ is also known as the expected heterozygosity (H_exp_). ${\hat{p}}_{\left(i,s\right)}$ is the coverage of a nucleotide at SNP(*s*) divided by the total coverage of *s* in collection (*i*). $n_{i}$ and $n_{j}$ are the number of mosquitoes sampled in collections *i* and *j*, and ${\hat{p}}_{s}$ is the coverage of a nucleotide at *s* in both *i* and *j* collections, divided by the total coverage of *s* in both *i* and *j* collections. The estimate of F_ST_ for *s* is:

$$F_{\mathrm{ST}}\left(s\right)=\frac{a_{s}}{a_{s}+b_{s}}$$

and, for an entire gene (*g*) with *m* SNPs, is:

$$F_{\mathrm{ST}}\left(g\right)=\frac{\sum_{s=1}^{m}a_{s}}{\sum_{s=1}^{m}\left(a_{s}+b_{s}\right)}$$

Graphing and statistical metrics were carried out in R-Bioconductor (Huber *et al.* 2015). Venn diagrams (Figure S2 and Figure S3) were prepared using the webtool Venny (Oliveros 2010).

### Data availability

All fastq files are available through the NCBI Sequence Read Archive (https://www.ncbi.nlm.nih.gov/sra) under accession numbers PRJNA258086 and SRP061709.

## Results

### Genetic diversity within Ae. aegypti collections

**Figure 1:**
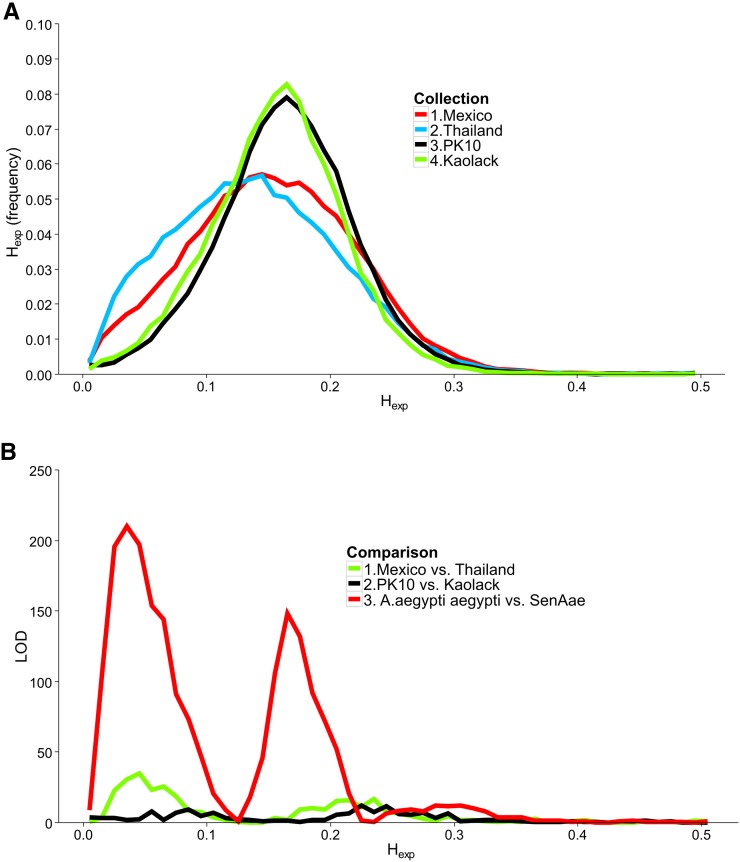
Expected heterozygosity (H_exp_) distribution for each collection. (A) *Aaa* Mexico (red line), *Aaa* Thailand (bright blue), *SenAae* PK-10 (black), and *SenAae* Kaolack (green). (B) H_exp_ distributions were compared with one another using a heterogeneity χ^2^ test of the number of genes in each 0.01 bin along the abscissa in the histogram. *Aaa* *vs.* *SenAae* comparisons (red line) have larger LOD differences in H_exp_ scores than *Aaa* *vs.* *Aaa* (green line) or *SenAae* *vs.* *SenAae* (black line).

Numbers of sequences in each of the six libraries appear in Table 1. To estimate the amount of genetic diversity within each collection, the percentage of polymorphic SNPs were calculated. The greatest percentage of polymorphism was observed in the PK10 (8.3%) and Kaolack (7.3%) collections. Figure 1A shows the distribution of H_exp_ in all four collections. The distribution of H_exp_ was shifted to the right in PK10 and Kaolack collections, whereas the H_exp_ distributions for the two *Aaa* collections were skewed toward lower values. Figure 1B shows the LOD scores associated with three heterogeneity χ^2^ analyses. Minor differences in H_exp_ distributions exist between Mexico and Thailand (green line), or between Kaolack and PK10 (black line). However, the differences in H_exp_ distributions between *Aaa* and *SenAae* were large and highly significant (red line).

**Table 1:** Numbers of monomorphic sites in a in the *Ae. aegypti* collection followed by the numbers of alternate nucleotides at polymorphic sites

		Number of Nucleotides/Indels						
Collection	Monomorphic	2	3	4	5	6	Total	% Polymorphic
PK10	30,229,087	2,580,103	148,547	9276	379	12	32,967,403	8.31%
Female	28,164,602	1,949,160	83,760	3976	131	6	30,201,634	6.74%
Male	28,324,103	1,988,143	90,424	4624	174	5	30,407,471	6.85%
Kaolack	30,259,280	2,250,995	121,064	7291	305	9	32,638,942	7.29%
Merida	28,592,543	1,459,996	42,017	1418	48	1	30,096,023	5.00%
Thailand	30,224,052	1,754,936	54,368	2035	72	2	32,035,463	5.65%
Female	26,580,423	1,119,509	25,775	816	30	1	27,726,553	4.13%
Male	27,166,304	1,222,100	30,373	979	35	0	28,419,790	4.41%

For example, 32,967,403 sites were analyzed in PK10. Of these, 30,229,087 of these were monomorphic, while 2,580,103 sites had two alternative nucleotides/indels, and 148,547 had three alternative nucleotides/indels. The percentage polymorphic is 100×[1 − (monomorphic/total)].

Table 2 shows a gene-wise comparison of H_exp_ in all six pairwise comparisons of collections, and between sexes, in PK10 and Thailand. H_exp_ in Mexico was greater that H_exp_ in Thailand at 67% of SNPs. H_exp_ in PK10 was greater than H_exp_ in the three other collections in 86–96% of SNPs. Interestingly, H_exp_ in Kaolack was less than H_exp_ in Thailand or Mexico.

**Table 2:** Expected heterozygosity (H_exp_) in *Ae. aegypti* collections

Comparison	Number of Genes	H_exp_ (1) > H_exp_(2)	H_exp_ (1) < H_exp_ (2)	% H_exp_ (1) > H_exp_ (2) (%)	Average Difference	Probability in Pairwise *t*-Test
(1) Mexico *vs.* (2) Thailand	15,735	10,628	5107	68	0.0018	≤2.2E–16
(1) PK10 *vs.* (2) Kaolack	15,735	15,211	524	97	0.0082	≤2.2E–16
(1) PK10 *vs.* (2) Mexico	15,735	13,650	2085	87	0.0054	≤2.2E–16
(1) PK10 *vs.* (2) Thailand	15,735	14,340	1395	91	0.0072	≤2.2E–16
(1) Kaolack *vs.* (2) Mexico	15,735	4509	11,226	29	0.0029	≤2.2E–16
(1) Kaolack *vs.* (2) Thailand	15,735	6841	8894	43	0.0010	≤2.2E–16
(1) PK10 female *vs.* (2) male	15,722	8499	7223	54	0.0008	≤2.2E–16
(1) Thailand female *vs.* (2) male	15,481	6910	8571	45	−0.0018	≤2.2E–16

For example, in comparing the Mexican and Thailand collections, 15,735 genes occurred in common between the two collections. In 10,628 of these genes, H_exp_ was greatest in the Mexican collection, while H_exp_ was greatest in 5107 genes in Thailand. H_exp_ was greatest in the Mexican collection in 68% of comparisons, and the average difference in H_exp_ between Mexican and Thailand collections was 0.0018, which was significantly greater than zero.

Next, H_exp_ values were ranked from smallest to largest, and genes with the lowest 1% of average H_exp_, and highest 1% of average H_exp_ were identified. Of a total of 490 genes in the lower 1% tail of the H_exp_ distributions, 376 occurred in only one collection, 80 occurred in two collections, 29 occurred in three collections, and five genes had low H_exp_ in all collections (Figure S2 and Table S1). AAEL008073-RA encodes a Hyaluronan/mRNA-binding protein that is known in humans to bind the mRNA of type-1 plasminogen activator inhibitor (PAI-1), and is thought to be involved in regulation of mRNA stability (Heaton *et al.* 2001). The second gene, AAEL014847-RA, encodes innexin 2 (AAEL014847, *inx2*), and is involved in postembryonic development in drosophilids (Holcroft *et al.* 2013). The third is the 28S rDNA gene, while AAEL007163-RA and AAEL014638-RA encode hypothetical proteins.

In the upper 1% (Table S2) (*n* = 326), 160 genes occurred in only one collection, 63 occurred in two collections, 58 occurred in three collections, while 44 genes were common in all collections (Figure S3). Half (22) coded for noncoding RNAs (ncRNAs), 21 of which were tRNAs, and one was the U2 spliceosomal RNA gene (AAEL017645). Three encode ankyrins—a family of adaptor proteins that help attach integral membrane proteins to the cytoskeleton; they have binding sites for the β-subunit of spectrin, and 12 families of integral membrane proteins. Ankyrins appear to be required to maintain the integrity of plasma membranes, and to anchor specific ion channels, ion exchangers, and ion transporters in the plasma membrane. Two encode mitochondrial membrane ATP synthase (F_1_F_0_ ATP synthase or Complex V) that produce ATP from ADP in the presence of a proton gradient across the mitochondrial membrane, and two encode lipid storage droplets surface binding protein 2(lsd2), which is essential for embryogenesis, and required for normal deposition of neutral lipids in the oocyte.

### Genetic differences among Ae. aegypti collections

**Figure 2:**
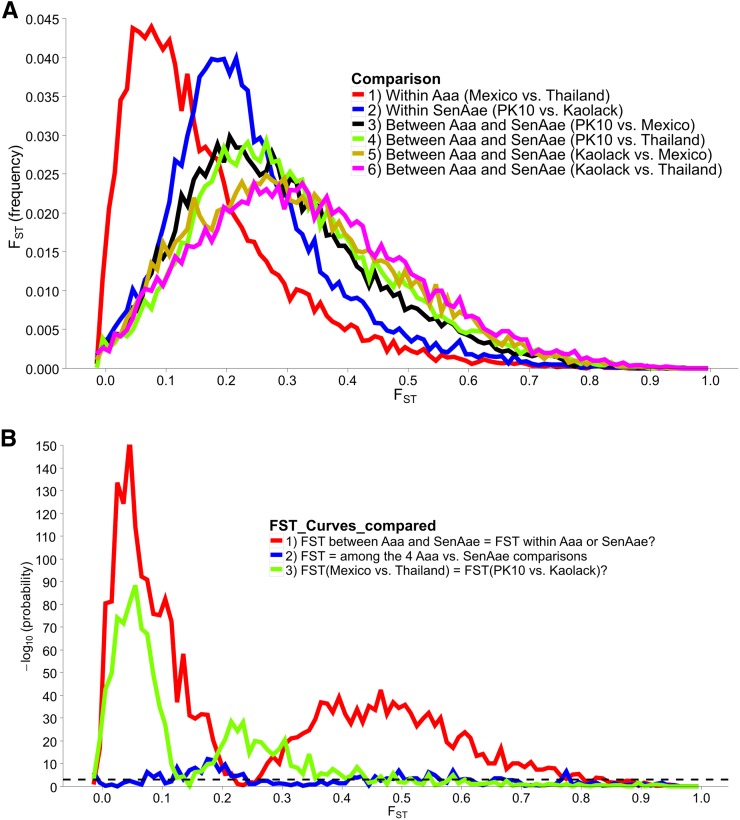
(A) Distributions of pairwise F_ST_ values among all six comparisons of the four collection sites. F_ST_ values between *Aaa* collections (red line) are skewed toward smaller values, while F_ST_ values between *SenAae* collections (blue line) were generally larger, and shifted to the right. All four F_ST_ distributions between *Aaa* and SenAae [*i.e.*, PK10 *vs.* Mexico (black), PK10 *vs.* Thailand (green), Kaolack *vs.* Mexico (tan), and Kaolack *vs.* Thailand (magenta)] had long right-hand tails, indicating larger F_ST_ values, and fewer small F_ST_ values. (B) F_ST_ distributions were compared with one another using a heterogeneity χ^2^ test of the number of genes in each 0.01 bin along the abscissa in the histogram. The red line is a comparison of F_ST_ between *Aaa* [red in (A)] and between *SenAae* [blue in (A)] with the four F_ST_ distributions curves [black, green, tan and magenta in (A)] between *Aaa* and *SenAae*. The left-hand portion of the red curve indicates a large and significant excess of small F_ST_ values within the *Aaa* collection, and within *SenAae* collections as compared with F_ST_ values between *Aaa* and *SenAae*. The right-hand region of the red curve indicates a large and significant deficiency of large F_ST_ values within *Aaa* collections, and within *SenAae* collections. The blue line in (B) is a comparison of the four *Aaa* *vs.* SenAae F_ST_ distributions (*i.e.*, PK10 *vs.* Mexico, PK10 *vs.* Thailand, Kaolack *vs.* Mexico, and Kaolack *vs.* Thailand). LOD values are never large, indicating that the four curves are similar. The green line is a comparison of the F_ST_ distributions within Aaa [red in (A)], and within *SenAae* [red in (A)]. The left-hand portion of the green curve indicates a large and significant excess of small F_ST_ values within *Aaa* collection as compared within *SenAae* F_ST_ values. The right hand region of the green curve indicates a large and significant deficiency of large F_ST_ values within *Aaa* collections.

To assess genetic diversity between pairs of collections, F_ST_ values were calculated for each of the six pairwise population comparisons, and the distribution of the F_ST_ values were plotted with a bin width of 0.01 using “hist(FST),breaks=seq(−0.05, 1, 0.01)” in R 3.1.0 (R Development Core Team 2014) (Figure 2). Each of these distributions are plotted individually in Figure S4, Figure S5, Figure S6, Figure S7, Figure S8, Figure S9, Figure S10, and Figure S11. The red line in Figure 2A is the distribution of pairwise F_ST_ values for the genes that occurred in common between Thailand and Mexico (*Aaa* collections), while the dark blue line is the distribution of pairwise F_ST_ for genes shared between PK10 and Kaolack (*SenAae* collections). The remaining curves are all comparisons between *Aaa* and *SenAae* (*i.e.*, Kaolack *vs.* Thailand, Kaolack *vs.* Mexico, PK10 *vs.* Thailand, and PK10 *vs.* Mexico). Table 3 lists the numbers of genes and SNPs in each pairwise comparison. Also listed are the mean, SD, median, and modal F_ST_ values, and the lower 5% and upper 95% F_ST_ cutoff values.

**Table 3:** Summary of F_ST_ distributions among all pairs of collections

Collections Compared	No. Genes	No. SNPs	Mean	Median	Mode	SD	Low 5%	High 95%
Thai sexes	15,345	1,109,671	0.015	0.007	−0.003	0.032	−0.013	0.068
PK10 sexes	15,442	1,882,229	0.030	0.014	0.000	0.047	−0.009	0.129
Mexico/Thailand	15,435	1,165,626	0.165	0.130	0.103	0.133	0.017	0.429
PK10/Kaolack	15,473	1,721,304	0.242	0.218	0.172	0.133	0.067	0.502
PK10/Mexico	15,453	1,266,724	0.289	0.267	0.185	0.155	0.074	0.582
PK10/Thailand	15,467	1,507,065	0.309	0.285	0.240	0.158	0.085	0.610
Kaolack/Mexico	15,452	1,665,639	0.316	0.297	0.298	0.168	0.073	0.616
Kaolack/Thailand	15,478	1,665,753	0.334	0.320	0.277	0.173	0.075	0.650

Four trends are evident. First, F_ST_ values between Thailand and Mexico are positively skewed, indicating that the majority of genes are similar between the two *Aaa* collections, despite the fact that the collection sites are ∼14,950 km apart (shortest arc distance). Second, pairwise F_ST_ values between the Kaolack and PK10 collections, which are ∼450 km apart, are shifted to the right, indicating that genetic differences between the two *SenAae* collections were greater than between the two *Aaa* collections. Third, F_ST_ values distributions between Senegal and the Thailand and Mexico collections are strongly shifted to the right, indicating large F_ST_ differences between *SenAae* and *Aaa*. LOD scores of a heterogeneity χ^2^ test are consistent with these observations (Figure 2B). Fourth, in examining Figure S4, Figure S5, Figure S6, Figure S7, Figure S8, Figure S9, Figure S10, and Figure S11, it is clear that the distribution of F_ST_ values between any two collections is not normally distributed, but rather is highly skewed. This is especially evident when comparing sexes in a collection (Figure S4 and Figure S5). Furthermore, the F_ST_ distribution shifts to the right as collections diverge genetically. For example, in comparing Mexico and Thailand collections (Figure S6), the mean F_ST_ (0.165 ± 0.130) is larger than the median (0.130), which in turn is larger than the mode (0.103). Furthermore, the curve underlying the lower 5% F_ST_ values, and the curve underlying the upper 95% F_ST_ values are asymmetrical in shape and distribution (Figure S6). Only when collections become extremely divergent (*e.g.*, Figure S8, Figure S9, Figure S10, and Figure S11) do the mean, median, and mode begin to converge. All of these inequalities arise because the genome-wide distribution of F_ST_ values is not normal. Therefore, centrality measures fail to capture the genome-wide variation in F_ST_, and summaries of genetic relationships based upon point estimates (mean, median, and mode) are misleading, and also miss the point of a population genomics analysis, which is to examine and compare patterns of variation across the entire genome.

### Genetic differences between sexes in Ae. aegypti collections

Our previous observations of chromosomal rearrangements on chromosome 1, along with the presence of the sex-determining locus (SDL) on chromosome 1 (Dickson *et al.* 2016), lead us to explore possible sex-specific chromosomal differences between dual replicate pools of males and females in the deep sequencing data in PK10 and Thailand. Roughly the same percentage of SNPS were polymorphic in the sexes in PK10 (6.74% in females and 6.85% in males) and Thailand (4.13% in females and 4.41% in males) (Table 1). H_exp_ in PK10 females was greater than H_exp_ in males at 54% of genes. H_exp_ in Thailand females was less than H_exp_ in males in 45% of genes.

**Figure 3:**
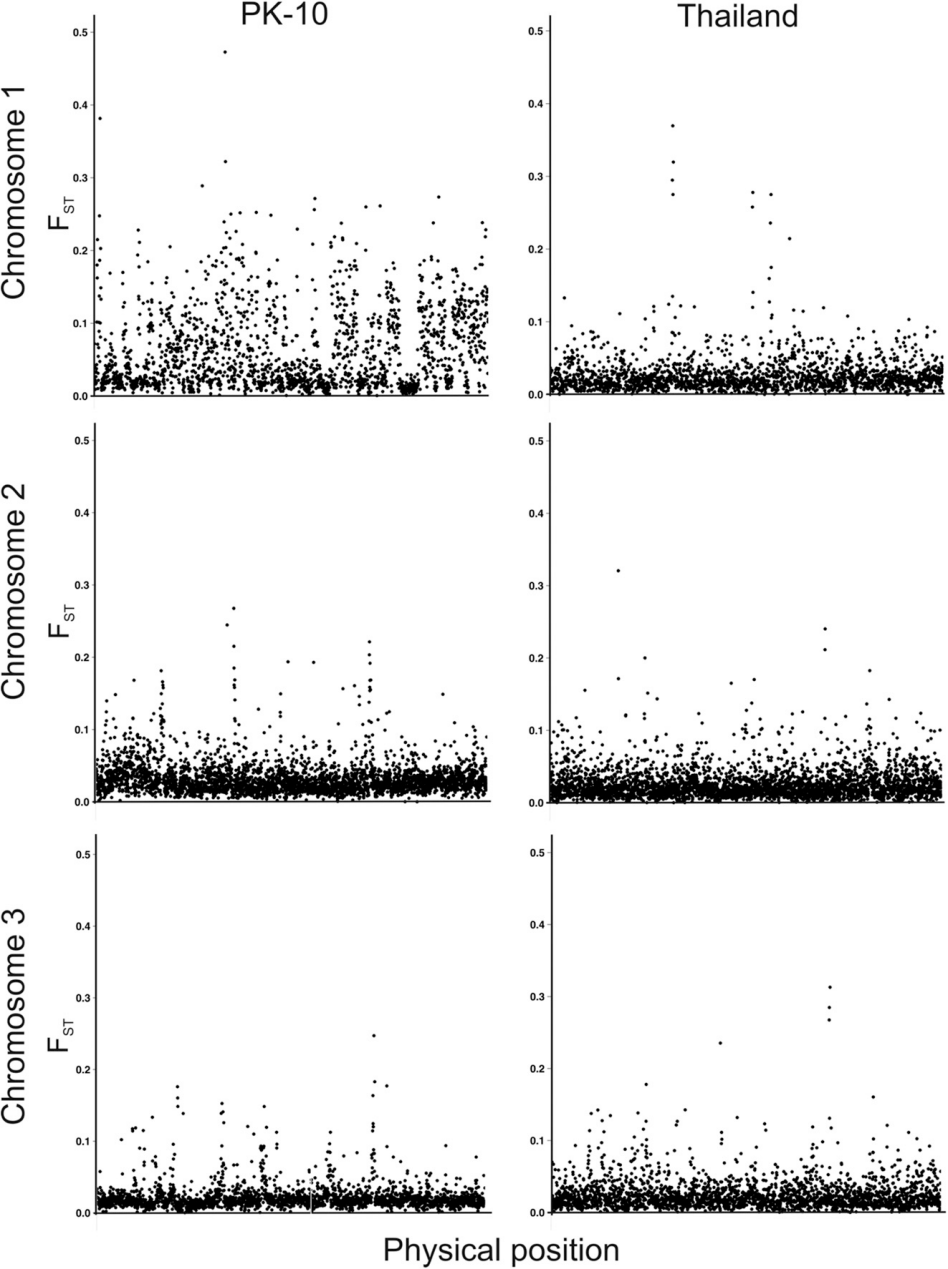
Comparison of F_ST_ values between sexes for genes that have been mapped across the three chromosomes in the PK10 (*SenAae*) and Thailand (*Aaa*) collections.

In addition to the F_ST_ sampling distributions between sexes (Figure S4 and Figure S5), F_ST_ values between sexes in both the PK10 and Thailand collections were mapped for each gene across the three chromosomes (Figure 3). Visual comparison of F_ST_ values on chromosome 1 in the PK10 and Thailand strains indicates that sex-specific differences were generally larger on chromosome 1 of the PK10 strain. Comparison of F_ST_ values on chromosome 1 between PK10 and Thailand were highly significant (pairwise-*t* = 26.9, d.f. = 1934, *p*-value = 1.15 × 10^−135^), and PK10 F_ST_ values were greater than Thailand F_ST_ values in 1387 of the 1935 genes compared (71.6%). These differences were distributed uniformly along chromosome 1, and did not appear to pile up in one or a few regions, as would be expected if the differences were associated with a single sex-determining locus. However, ∼50% of the *Ae. aegypti* genome remains unannotated, and recent work on mapping with the same markers used in this study revealed many cases where genes were misassembled (Juneja *et al.* 2014). Thus, it is possible that large F_ST_ values associated with markers on chromosomes 2 and 3 could actually map to chromosome 1. On chromosome 2, F_ST_ values were also significantly greater in PK10 (*t* = 12.6, d.f. = 3391, *p*-value = 1.31 × 10^−35^), and PK10 F_ST_ values were greater than Thailand F_ST_ values in 2110 of 3392 genes compared (62.2%). In contrast, on chromosome 3, F_ST_ values were actually significantly lower between PK10 sexes (*t* = −6.50, d.f. = 2354, *p*-value = 9.64 × 10^−11^), and PK10 F_ST_ values were greater than Thailand F_ST_ values in 1050 of 2355 genes compared (44.6%).

**Figure 4:**
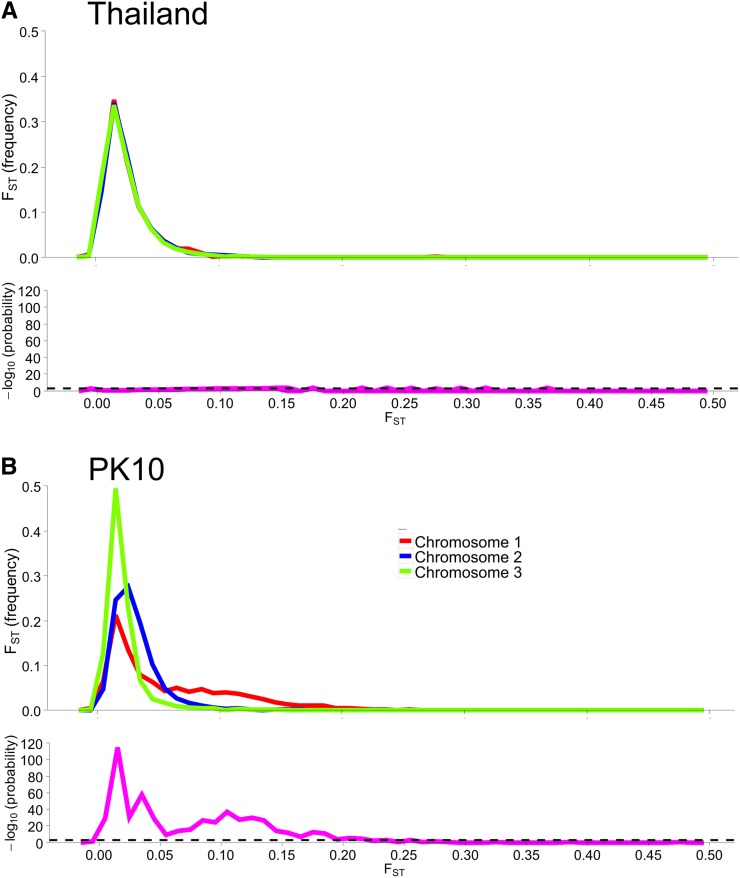
Distribution of F_ST_ and LOD values between sexes plotted on each chromosome. F_ST_ distributions were compared with one another using a heterogeneity χ^2^ test of the number of genes in each 0.01 bin along the abscissa in the histogram. (A) F_ST_ distributions are very similar among all three chromosomes in the Thailand collection. The magenta line indicates the LOD value across the chromosomes, while the black dashed line is the LOD = 3 cutoff. LOD values never exceed the LOD = 3 cutoff. (B) F_ST_ distributions differ among all three chromosomes in PK10 (*SenAae*). LOD values across the chromosomes exceed the LOD = 3 cutoff across all three chromosomes.

Figure 4 suggests greater differences in SNP frequencies between sexes on chromosome 1 within PK10 than among genes on all three chromosomes within Thailand. To test this further, the distribution of F_ST_ values between sexes was plotted for all three chromosomes (Figure 4). The distribution of F_ST_ values are very similar among all three chromosomes in the Thailand collection (Figure 4A). All three distributions were compared against one another using a 3 × 2 heterogeneity χ^2^ test of the number of genes in each 0.01 bin. None of the values crossed the LOD = 3.0 threshold. In contrast, the distribution of F_ST_ values are different between sexes on all three chromosomes in PK10 (Figure 4B). Specifically, the F_ST_ distribution for chromosome 1 is strongly shifted to the right, while, for chromosome 2, the distribution is slightly shifted to the right, and for chromosome 3, the distribution is positively skewed. F_ST_ from 0 to 0.2 cross the LOD = 3.0 threshold. Thus, while markers on all three chromosomes are at similar frequencies in the two sexes in the Thailand *Aaa* populations, they are at strikingly different frequencies between PK10 sexes.

**Figure 5:**
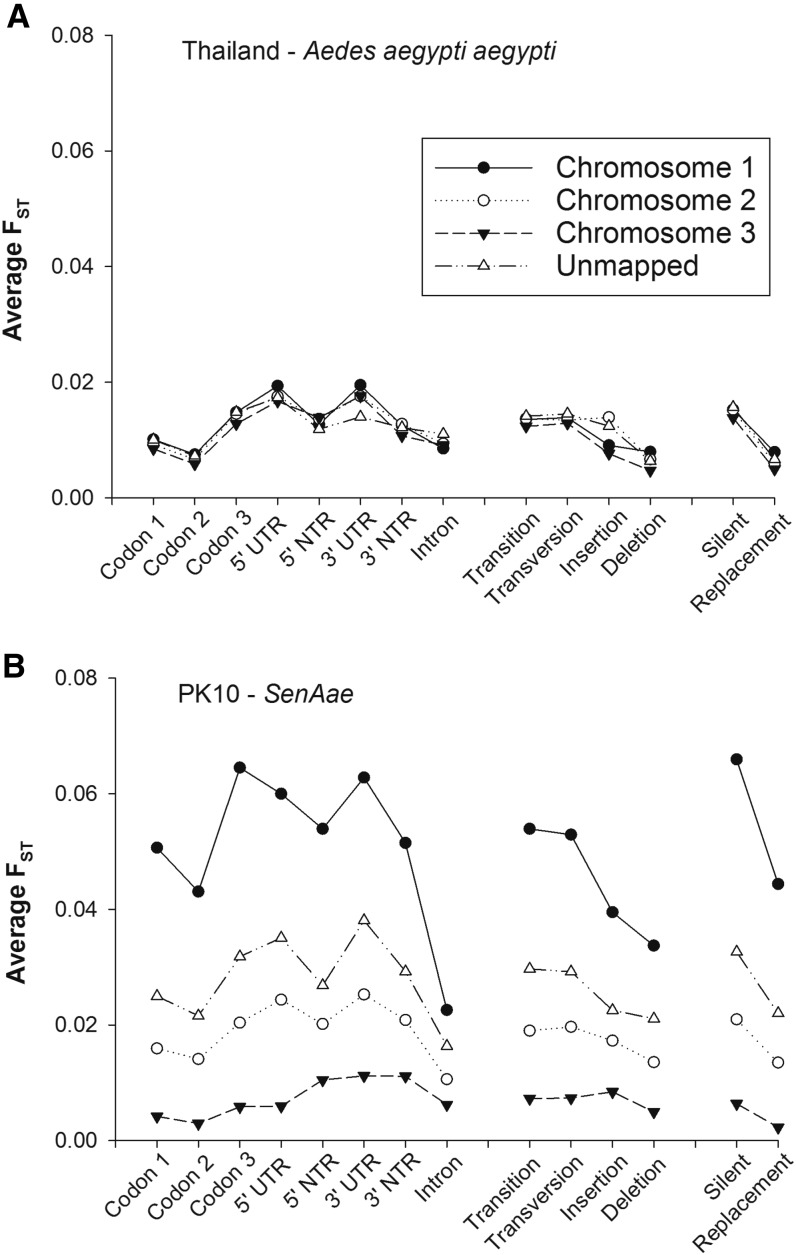
Average sex-specific F_ST_ values for SNPs in different regions of a gene. Values were plotted by chromosome in (A) Thailand (*Aaa*) females *vs.* males, and (B) PK10 (*SenAae*) females *vs.* males.

Figure 5 repeats the analyses on Figure 3 and Figure 4, but compares F_ST_ values according to the gene region in which the SNP occurred including codon positions 1, 2, and 3, introns, 5′UTR, and 600 bp on the 5′NTR, 3′UTR, and 600 bp on the 3′NTR. When comparing sexes in Thailand, F_ST_ values were greatest among SNPs in the 5′ and 3′ UTRs, and least among SNPs in the second codon position. Values were virtually equivalent on all three chromosomes, and including unmapped regions. F_ST_ values were also compared among the four types of mutations (transition, transversion, insertion, and deletion) giving rise to the SNP. F_ST_ values were equivalent between transitions and transversions, slightly lower for insertions, and lowest for deletions. Again, F_ST_ values were virtually equivalent on all three chromosomes and in unmapped regions. F_ST_ values were finally compared among SNPs occurring in codons that encoded a silent or replacement substitution. While F_ST_ values among silent SNPs were higher than among replacement SNPs, F_ST_ values were virtually equivalent among all three chromosomes and including unmapped regions.

In contrast, when comparing sexes in PK10, F_ST_ values between sexes were greatest among SNPs in the third codon position and in the 5′ and 3′ UTRs, and least among SNPs in the second codon position and introns. Values differed greatly among chromosomes and unmapped regions. The largest F_ST_ values occurred on chromosome 1 among all eight gene regions. The second largest F_ST_ values occurred on chromosome 2 among all eight gene regions, while the smallest F_ST_ values occurred on chromosome 3. The unmapped markers represent an average of all three chromosomes. Similar trends are evident among the transition, transversion, insertion, and deletion SNPs, and among SNPs encoding silent and replacement substitutions.

## Discussion

In keeping with earlier studies, the percentage of polymorphic SNPs and H_exp_ was greatest in the African *Ae. aegypti* collections. This is consistent with the idea that *Ae. aegypti* arose as a species somewhere in the African continent (Tabachnick 1991, 1979). A total of 490 genes occurred in the lower 1% tail of the four H_exp_ distributions, and most (77% = 376/490) occurred in only one collection, while only five genes (1%) had low H_exp_ in all collections (Figure S2). In contrast, 324 genes occurred in the upper 1% tail of the four H_exp_ distributions, and a smaller percentage (49% = 160/324) occurred in only one collection, while 44 genes (13.8%) were common in all four collections (Figure S3). Many of the genes in Table S1, have hundreds or thousands of SNPs. This probably reflects a pattern of neutral evolution, wherein a forward mutation rate generates new SNPs independently among genes and among SNPs within genes. In the context of this project it would be assumed that a mutation (SNP) arose only once in single collections (infinite alleles model), and drift would cause the majority of these to become extinct in a few generations, depending upon the effective population sizes. In contrast, the genes that occur in common among the four collections are probably maintained at a low H_exp_ by purifying selection. For example, 24 of the low H_exp_ genes encode rRNA genes that would be expected to be under strong purifying selection, and homogeneous due to the molecular drive operating in the rDNA cistron (Table S2).

By comparison, the 324 genes that had a high H_exp_ (Table S3) do not follow a pattern consistent with neutral evolution, and, instead, are either not constrained by purifying selection, or are subject to some form of diversifying selection. Curiously 35.4% (115/325) of these encode either tRNAS(86), tRNA pseudogenes(19), or, while annotated as tRNAs in VectorBase, 10 could not be confirmed with tRNAscan-SE/ (Schattner *et al.* 2005). Forty-four high H_exp_ genes (14%) occurred in common among all collections (Figure S2). Half of these 44 genes coded for noncoding RNAs (ncRNAs), 21 of which were tRNAs, and one was the U2 spliceosomal RNA gene (AAEL017645). This suggests a common lack of purifying selection, or diversifying selection, acting upon these genes in all collections.

*Ae. aegypti* has 962 annotated nuclear-encoded transfer RNA (tRNA) genes arranged in 61 genomic clusters, with 111 predicted as pseudogenes (Behura and Severson 2011; Lawson *et al.* 2007; Schattner *et al.* 2005). Members of the tRNA functional class were present in both the high H_exp_ (116 tRNAs) and low H_exp_ (22 tRNAs) (Table S1 and Table S2). The majority of low H_exp_ occurred in *SenAae* (Table S2), while the highest H_exp_ occurred in all collections. Of these, 62% had anticodons predicted to have preferred codon usage (Behura and Severson 2011). Curiously, 32% (7/22) of the low H_exp_, and 19% (19/113) of high H_exp_ tRNAs, were predicted to be pseudogenes (Schattner *et al.* 2005).

Why do aedine mosquitoes have many tRNA isoacceptor gene copies? Moreover, why is a subset of pseudogenes highly conserved? Varied codon usage, which would partially contribute to multiple tRNA copy numbers, has been suggested to be pertinent to specific metabolic pathways (Behura and Severson 2011), and could affect stage-specific expression patterns. Moreover, tRNAs also participate in regulatory activity, as tRNA fragments have been found to regulate sperm maturation in mammals (Sharma *et al.* 2016). Supportive evidence in aedine mosquitoes has also been described; for example, there is evidence for general RNA transfer in seminal fluid (Alfonso-Parra *et al.* 2016). Moreover, tRNA fragments (15–30 nt) have been isolated from small RNA (sRNA) sequencing pools (Hess *et al.* 2011). Although the mechanistic details of possible tRNA regulation are yet to be elucidated in mosquitoes, these studies, coupled with the presence of over 100 tRNA genes showing high H_exp_ values, is consistent with the hypothesis that tRNA polymorphisms may arise during speciation.

F_ST_ differences were most pronounced between the two *SenAae* collections, and between *SenAae* and *Aaa*. F_ST_ values were ranked from smallest to largest, and genes with the lowest 1% of F_ST_ are listed in Table S3, and those with the highest 1% of F_ST_ appear in Table S4. Fifty genes occurred within the lowest 1%. Of these, none occurred only once or twice, 29 occurred three times, none occurred four times, 15 occurred in five comparisons, and six occurred in all comparisons, suggesting these are highly conserved across populations. AAEL017730 was predicted to encode a novel miRNA, but was not similar to any known hexapod miRNAs (Griffiths-Jones *et al.* 2008). AAEL006000-RC is predicted to encode cytochrome *c* oxidase polypeptide 7A1—one of the nuclear-coded polypeptide chains of cytochrome *c* oxidase, the terminal oxidase in mitochondrial electron transport. AAEL008026-RA encodes an MADF (myb/SANT-like domain in Adf-1), where the SANT domain (acronym for Swi3, Ada2, N-Cor, TFIIB) is a highly conserved motif. In *Drosophila*, Adf-1 encodes a transcription factor that binds the promoters of a diverse group of genes (England *et al.* 1992). AAEL016955-RA encodes a tRNA-Asp, while AAEL006284-RA and AAEL006943-RA encode hypothetical proteins.

Similarly, the upper 1% of F_ST_ values were interrogated for each of the six pairwise comparisons (Table S4). Of 48 genes, only CYP6P12 (AAEL014891) occurred in all comparisons. The remaining genes all appeared three times. CYP6P12 is a major insecticide detoxification gene (Saavedra-Rodriguez *et al.* 2014). Several other genes known to be involved in insecticide resistance occurred in the upper 1% of F_ST_ values. AAEL012491-RA encodes a different CYP6P12, while four epsilon glutathione-S-transferases (2, 4, 6, and 7) appear in this list. This in consistent with an hypothesis that insecticidal pressure [very high in Mexico (Flores *et al.* 2013) and Thailand (Bosio *et al.* 2005), very low or nonexistent in Senegal (Sylla *et al.* 2013)] has promoted directional selection, and caused large differences in the frequencies of SNPs. Reductions in heterozygosity usually accompany directional selection with insecticides. The average H_exp_ among the four epsilon GSTs was PK10 (0.166), Kaolack (0.210), Mexico (0.061), and Thailand (0.023), but H_exp_ among the CYP6P12 was: PK10 (0.130), Kaolack (0.140), Mexico (0.166), and Thailand (0.038).

In the process of completing this study, we learned an important technical aspect of next generation sequencing (NGS) when applied to species (such as *Ae. aegypti*) with large genomes that contain a high percentage of repetitive DNA (Warren and Crampton 1991). Early on in this investigation, we tried to perform the same NGS analyses but without exon enrichment. From 23 to 37 × 10^6^ sequences were recovered in each of the 12 libraries; however, codon regions were detected in <5% of all recovered sequences. Reassociation kinetics of the *Ae. aegypti* genome (Warren and Crampton 1991) indicated that 40% of the genome consisted of fold-back, highly repetitive, and middle repetitive DNA. Perusal of VectorBase indicates that much of the remaining “unique” DNA consists of transposable elements. Most importantly, analysis of these unenriched datasets provided no resolution of subspecies (Figure 1 and Figure 2), nor did it identify many differences between sexes (Figure 4 and Figure 5).

The results of this study suggest that genes from throughout the genome are similar between *Aaa* from two geographically distinct locations. *SenAae* genomes also differed from one another to a large extent in the present study, but, more notably, differ greatly between *Aaa* and *SenAae*. We have yet to determine how *SenAae* are related to East African *Aaf*, but this same pattern was evident in the recent study of worldwide collections of *Ae. aegypti* (*s.l*.) by Evans *et al.* (2015) using a 50,000 SNP-Chip. In particular, their principal components analysis (PCA) of collections from Africa, Asia and the Pacific, and the Americas (Figure 3B in Evans *et al.* 2015) showed that the five Americas collections formed a very tight overlapping cluster distinct from, but on the same side of the PCA, as the four Asia and the Pacific collections, which formed three distinct clusters. In contrast, African collections (including two *SenAae* but neither PK10 nor Kaolack) formed very large clusters. One apparent difference between the PCA and results in the present study is that Thailand and Mexico collections in the PCA appear to be more distinct than in our comparison of F_ST_ distributions. However, our results do not necessarily contradict those of Evans *et al.* (2015). First, collection sites in Thailand differed between the two studies. Second, referring to Panel B of the PCA in Figure 3 of Evans *et al.* (2015), the Thailand (purple squares) and Tapachula (gold diamonds) collections are extremely close along the PC1 axis that accounts for 13.2% of the variation in SNP frequencies. Thailand and Tapachula only separate along PC2, which accounts for only 4.5% of the variation. Third, PC1+PC2 account for only 17.7% of the overall variation, indicating that 82% of the variation in SNP frequencies is unexplained by the first two PCs. Fourth, PCA is an analysis of individuals, while our analysis of F_ST_ is based on collections of individuals. Evans *et al.* (2015) does not indicate how much variation exists among individuals in a collection (this appears for our data in Figure 1). Could within-collection variation among individuals account for a large part of the 82% of unexplained variance? Finally, Figure S4, Figure S5, Figure S6, Figure S7, Figure S8, Figure S9, Figure S10, and Figure S11 show that summary of genetic relationships based upon point estimates (mean, median, mode, and variance) is misleading.

These patterns in genic diversity correlate with the reproductive isolation patterns noted in our earlier study (Dickson *et al.* 2016). In that study, attempts to construct F_1_ intercross families between *Aaa* laboratory strains and *SenAae* failed due to poor F_1_ oviposition and low F_2_ egg-to-adult survival. Backcrosses were performed to test for postzygotic isolation patterns consistent with Haldane’s rule modified for species, like *Aedes*, that have an autosomal SDL (Presgraves and Orr 1998). However, these crosses were inconclusive with regards to Haldane’s rule. Instead, basic cytogenetic analyses and FISH experiments revealed many rearrangements between *SenAae* and *Aaa*. Linkage analysis of the SDL, and the white-eye locus, identified a likely chromosomal rearrangement on chromosome 1. Another rearrangement was close to the centromere on the p arm of chromosome 2. Two overlapping pericentric inversions were found on chromosome 3, or an insertion of a large fragment into the 3q arm. The reproductive incompatibility observed within *SenAae*, and between *SenAae* and *Aaa*, may be generally associated with chromosome rearrangements on all three chromosomes, and specifically caused by pericentric inversions on chromosomes 2 and 3. Chromosomal rearrangements have frequently been associated with speciation in taxa, including mosquitoes (Kitzmiller 1963). Pericentric inversions are only rarely detected within animal species, but can be abundant among species (Coyne and Orr 2004; Orr and Coyne 1989). Acentric fragments and dicentric “bridge” chromosomes arise when recombination occurs in parents that are heterozygous for pericentric inversions. These, in turn, yield aneuploid gametes and inviable zygotes.

We hypothesize that cryptic *Ae. aegypti* taxa exist in West Africa, or that new subspecies of *Ae. aegypti* are arising in West Africa. We propose that the large genic differences between *Aaa* and *SenAae* documented in the present study have accumulated over a long period of time, possibly following the occurrence of chromosome rearrangements in allopatric populations that subsequently caused the taxa to become reproductively isolated when populations became sympatric. It may be that PK10 represents a collection of at least two fully, or partially, reproductively isolated taxa. If so, it is curious that the largest differences between PK10 females and males occur on the sex autosome 1 (Figure 3). Sex determination is governed by a dominant male-determining “M” allele in the sex determination locus on chromosome 1 in *Ae. aegypti*. The SDL gene “Nix” has recently been identified. *Nix* exhibits persistent M linkage and early embryonic expression (Hall *et al.* 2015); however differences between PK10 females and males were not limited to one region of chromosome 1. It has been proposed that sexual selection may drive the more rapid evolution of male-expressed genes (Wu and Davis 1993; Wu *et al.* 1996; Coulthart and Singh 1988), as well as variation in expression of male-expressed genes (Harrison *et al.* 2015). More rapid male evolution would also explain Haldane’s rule because hybrid male sterility would occur before hybrid female sterility. Experiments in *Drosophila* suggest that faster male evolution causes Haldane’s rule for sterility (Hollocher and Wu 1996; Harrison *et al.* 2015; True *et al.* 1996). Unfortunately there is no information to suggest that male-expressed genes occur primarily on chromosome 1 or within the region containing *Nix* (Hall *et al.* 2015).

Herein, we have demonstrated large amounts of genic differentiation between *SenAae* and *Aaa* populations outside Africa, and earlier have provided evidence for chromosome rearrangements (Dickson *et al.* 2016). These observations are important because there is currently a great deal of interest in manipulating or eliminating *Ae. aegypti* populations on a global scale (Zhang *et al.* 2015; Rasic *et al.* 2014). The presence of cryptic *Ae. aegypti* species could negatively impact this effort. There is a great need to define the geographic distributions of chromosome rearrangements that are presumably associated with these cryptic *Ae. aegypti* taxa or subspecies.

## Supplementary Material

Supplemental material is available online at www.g3journal.org/lookup/suppl/doi:10.1534/g3.116.036053/-/DC1.
