# Supplementary figures and images for: Exon-Enriched Libraries Reveal Large Genic Differences Between Aedes aegypti from Senegal, West Africa, and Populations Outside Africa
Source: G3 (Bethesda). 2016 Dec 19;7(2):571–82. doi: 10.1534/g3.116.036053 (PMC5295602; doi:10.1534/g3.116.036053)

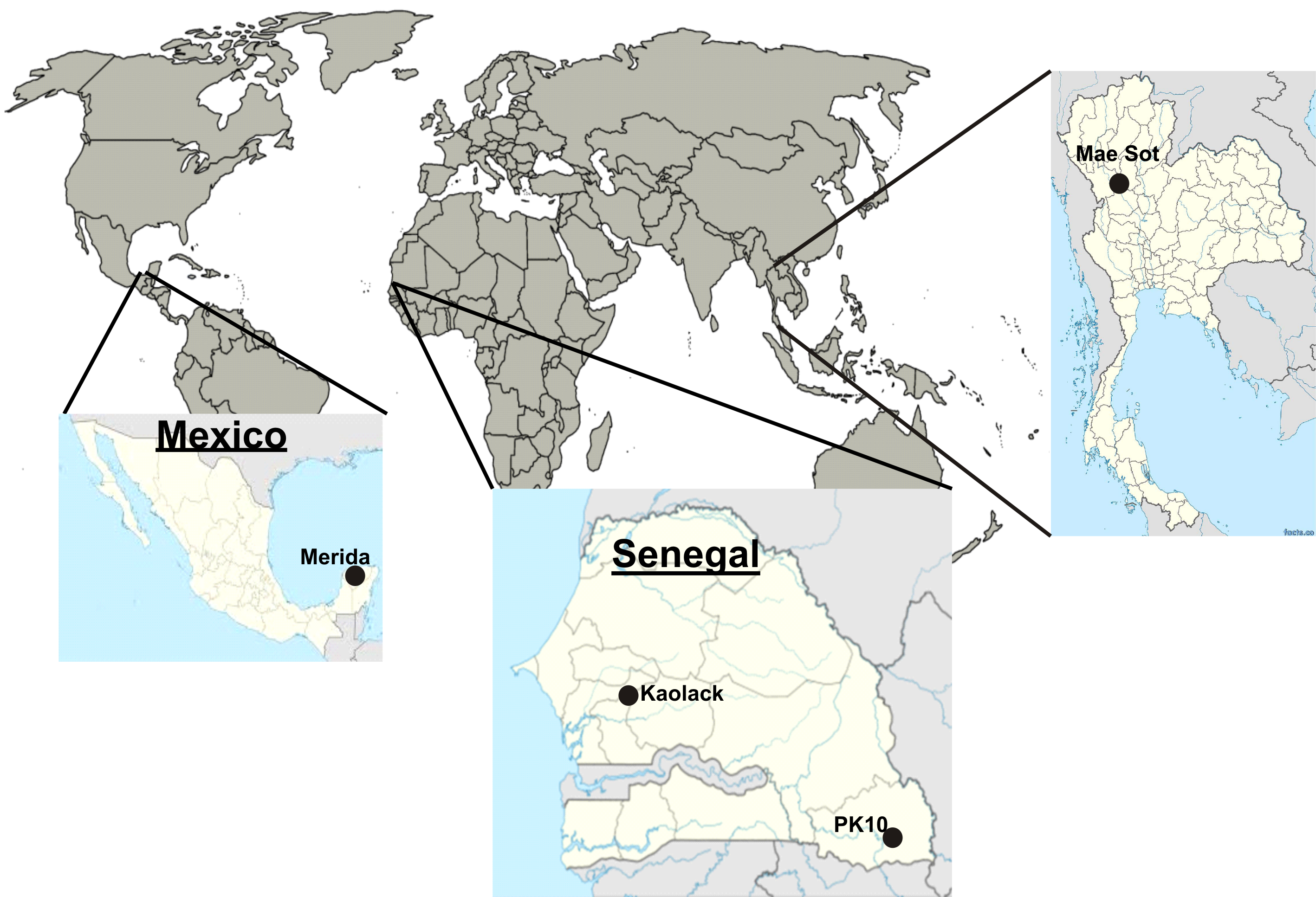

Supplement: Supplementary file 1 [file 571FigureS1.tif]

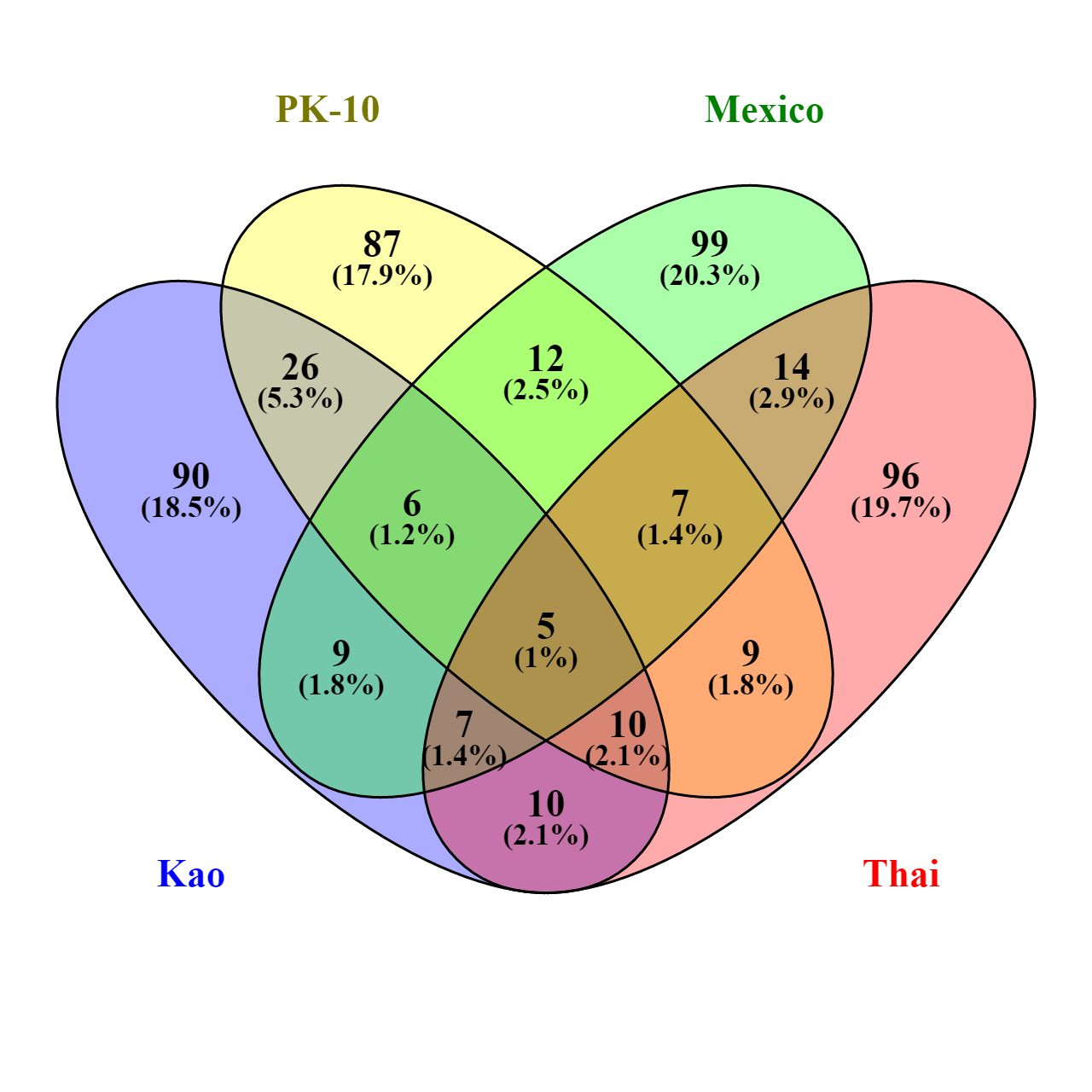

Supplement: Supplementary file 2 [file 571FigureS2.tif]

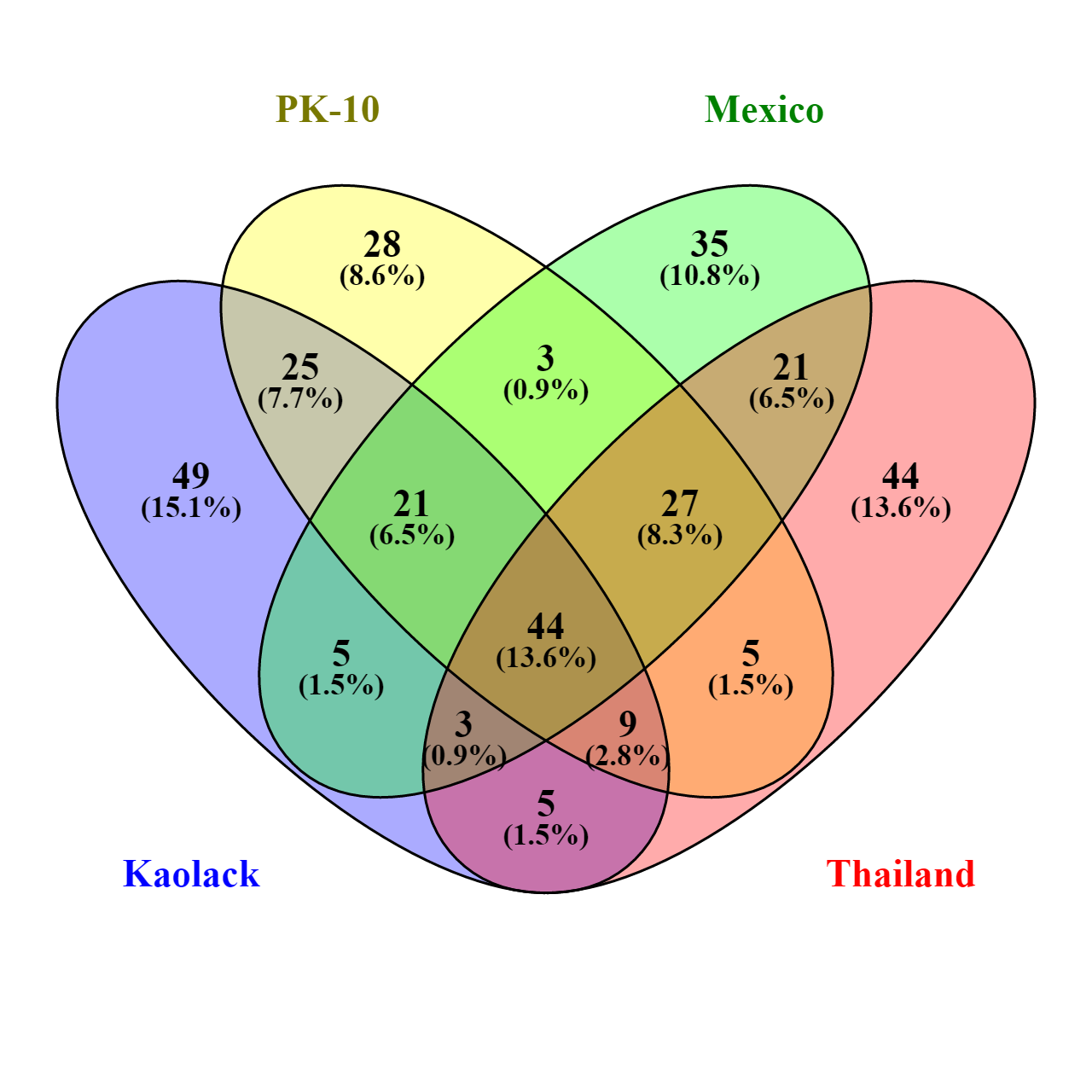

Supplement: Supplementary file 3 [file 571FigureS3.tif]

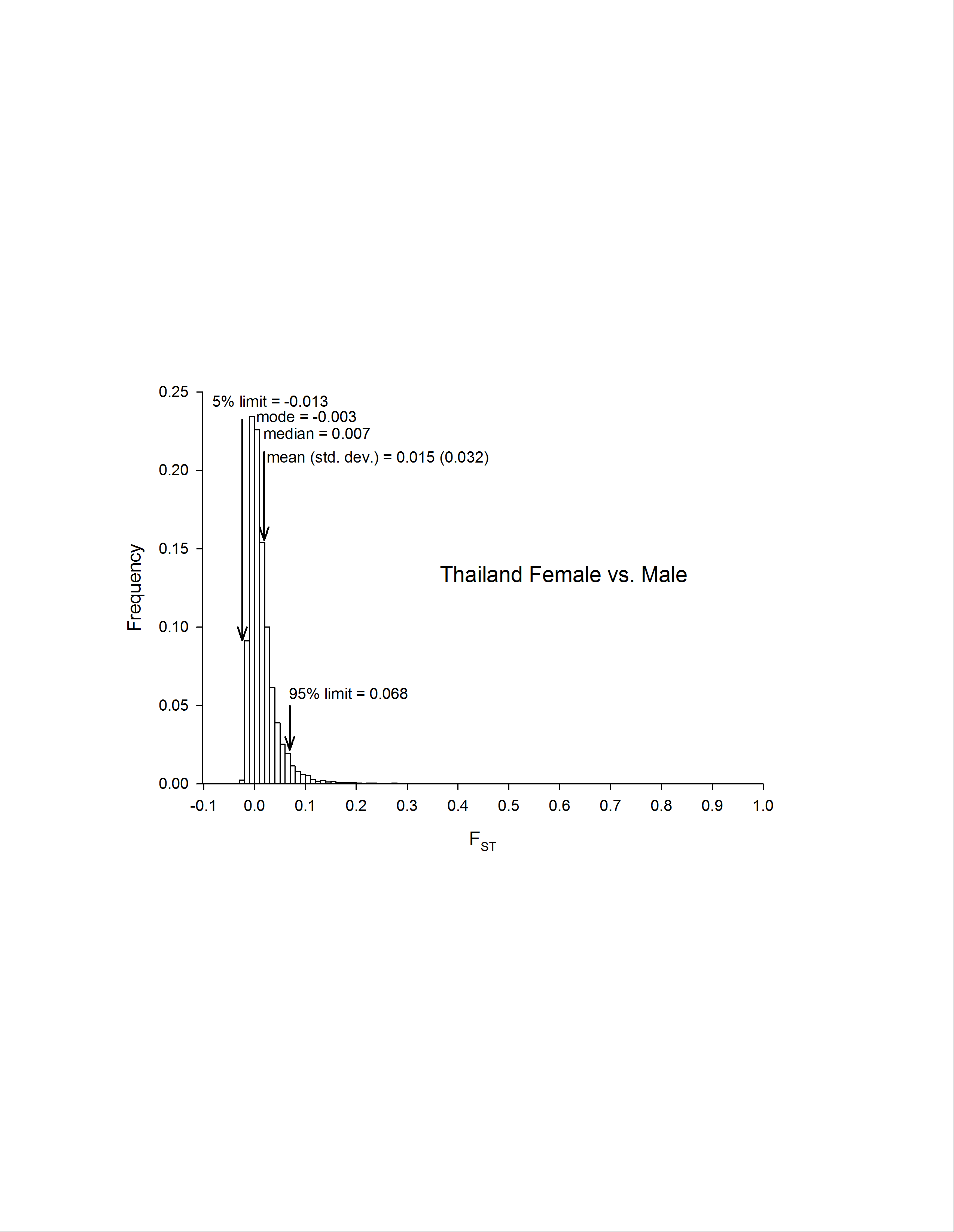

Supplement: Supplementary file 4 [file 571FigureS4.tif]

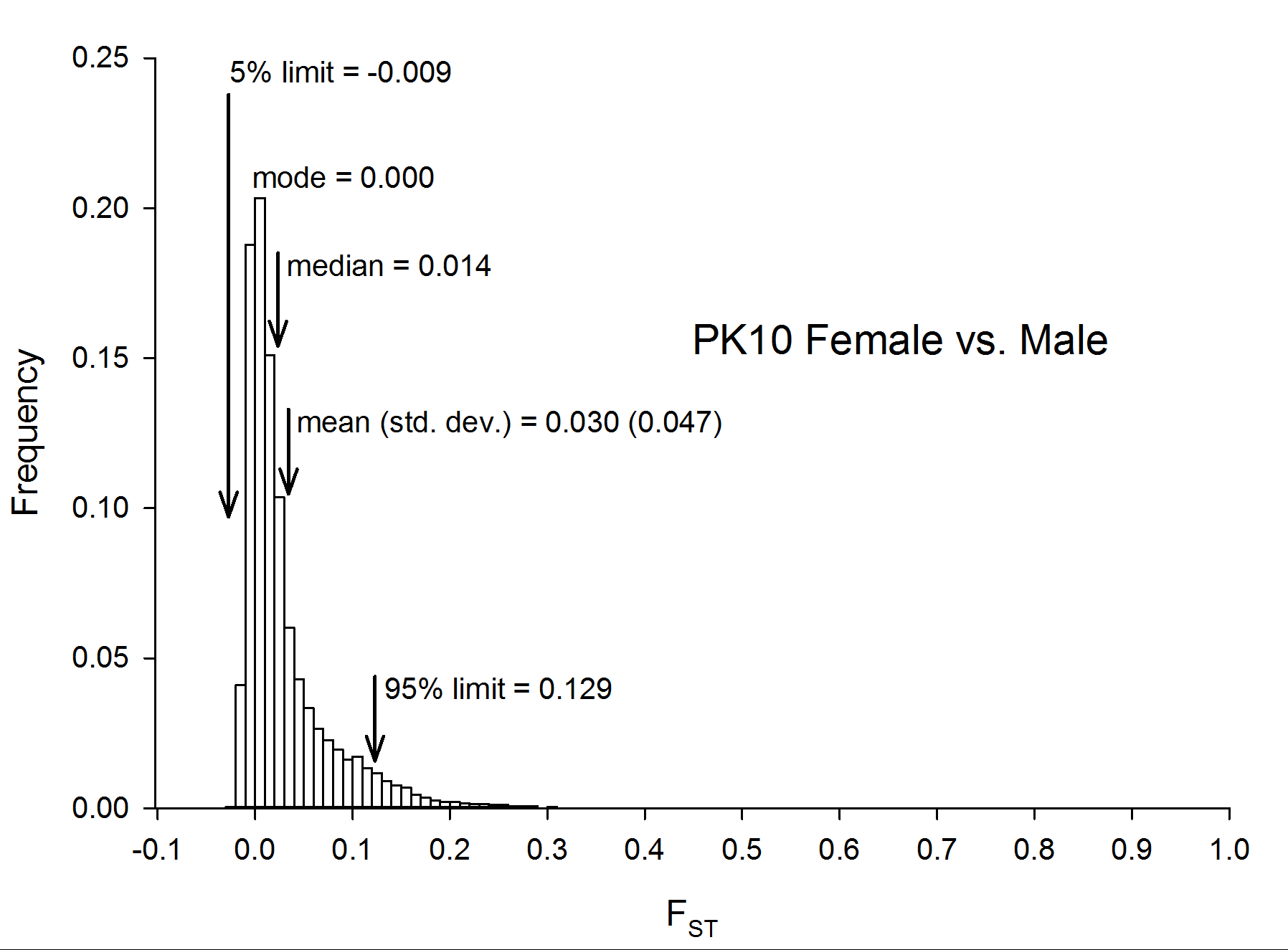

Supplement: Supplementary file 5 [file 571FigureS5.tif]

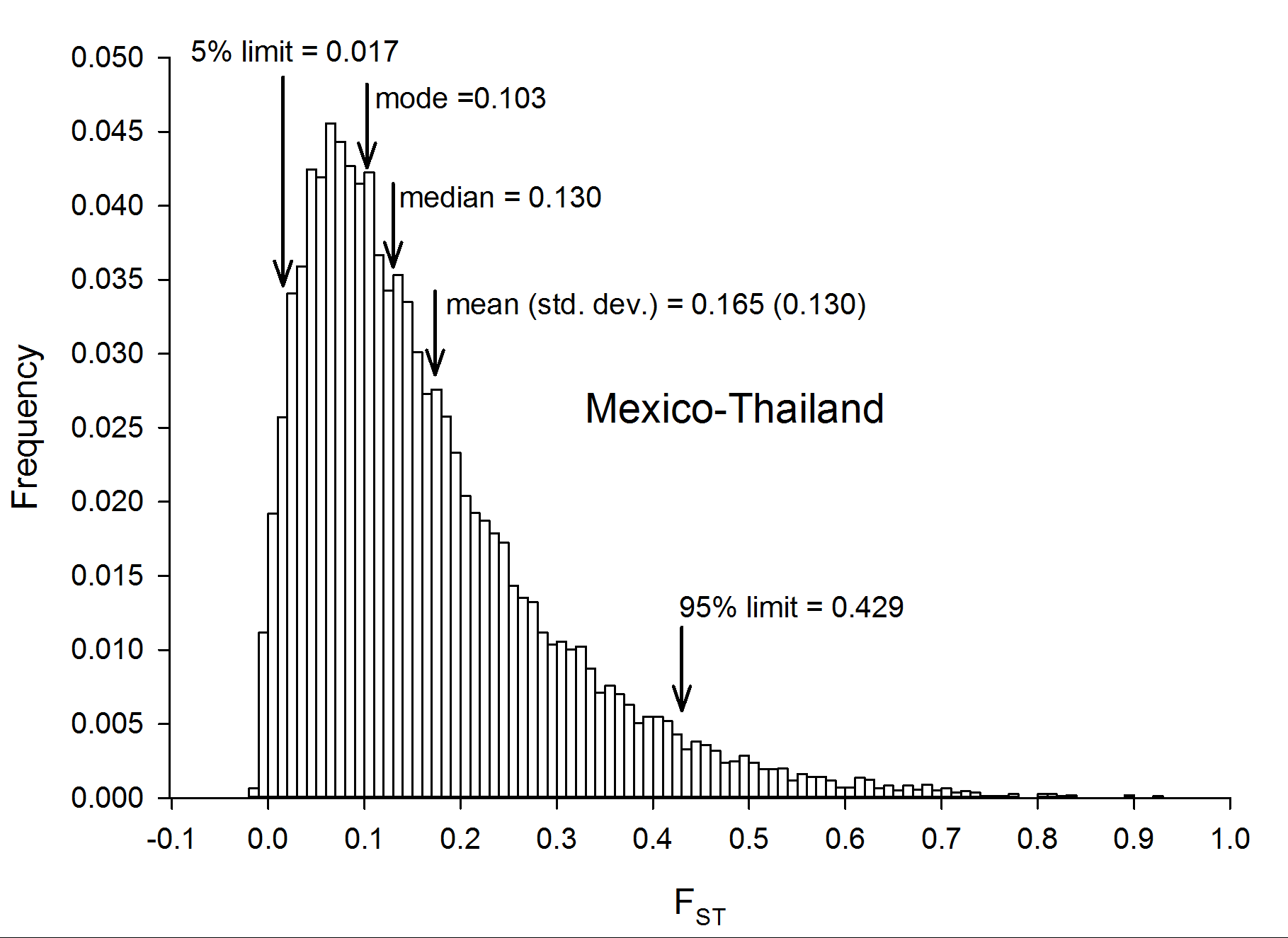

Supplement: Supplementary file 6 [file 571FigureS6.tif]

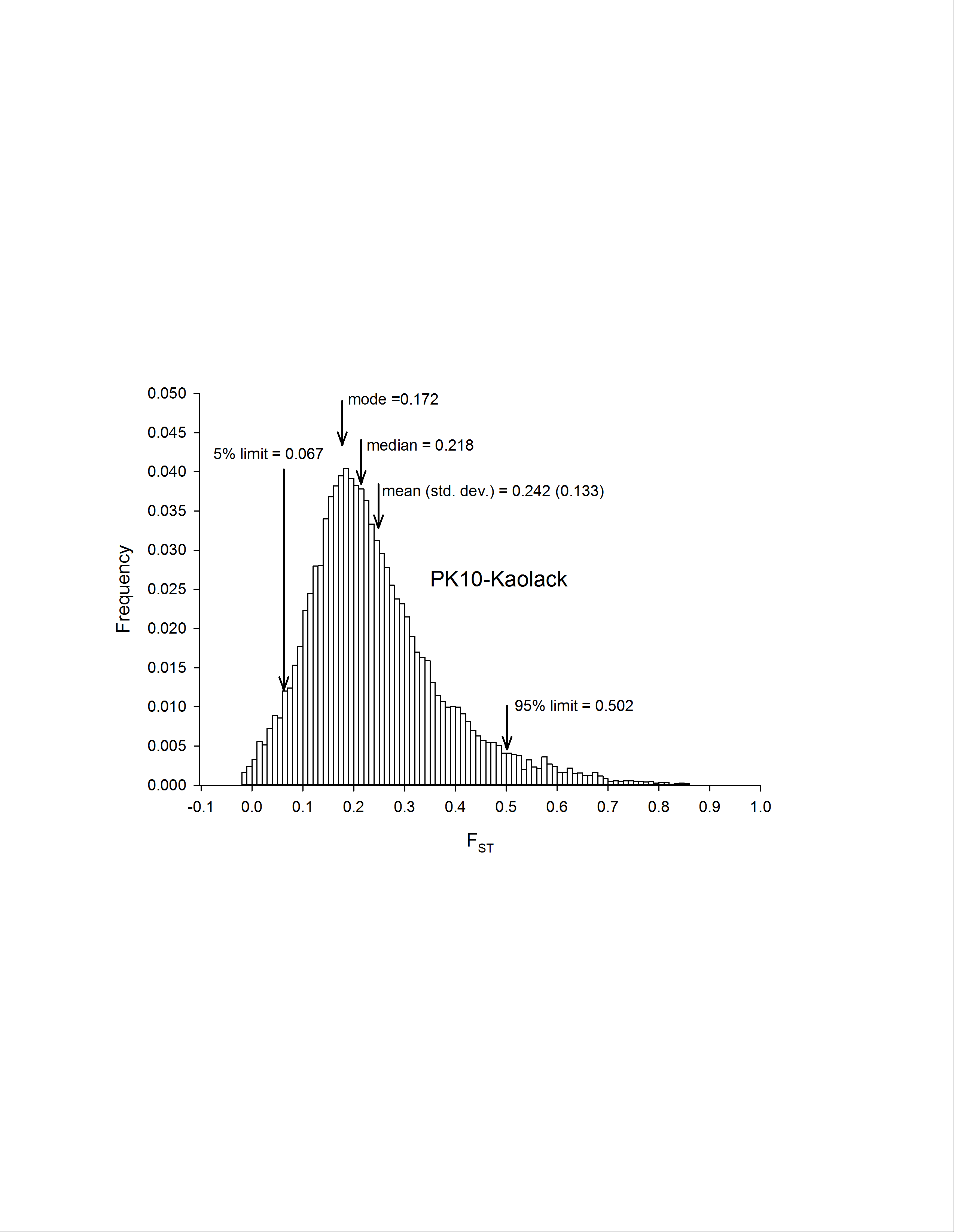

Supplement: Supplementary file 7 [file 571FigureS7.tif]

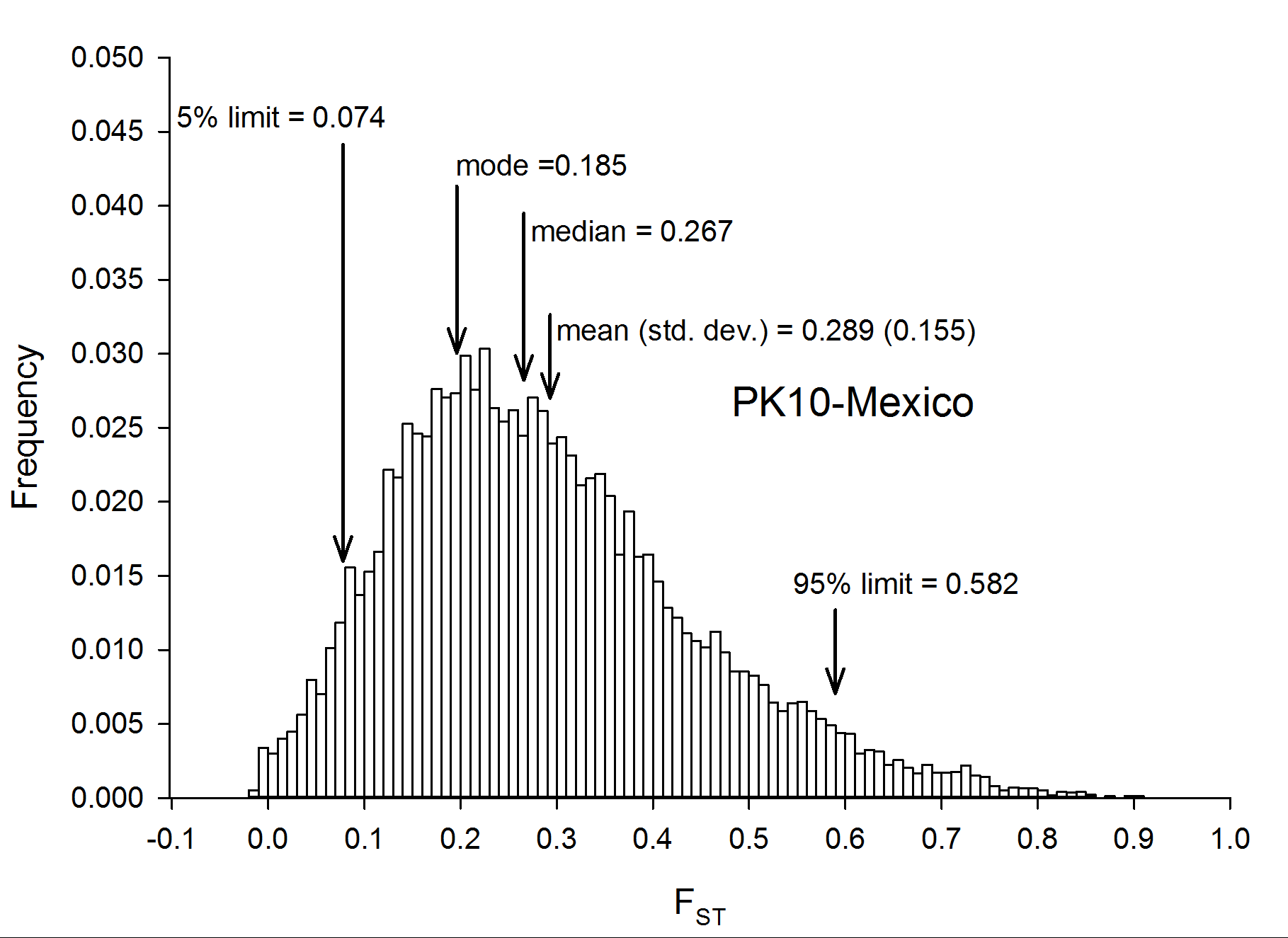

Supplement: Supplementary file 8 [file 571FigureS8.tif]

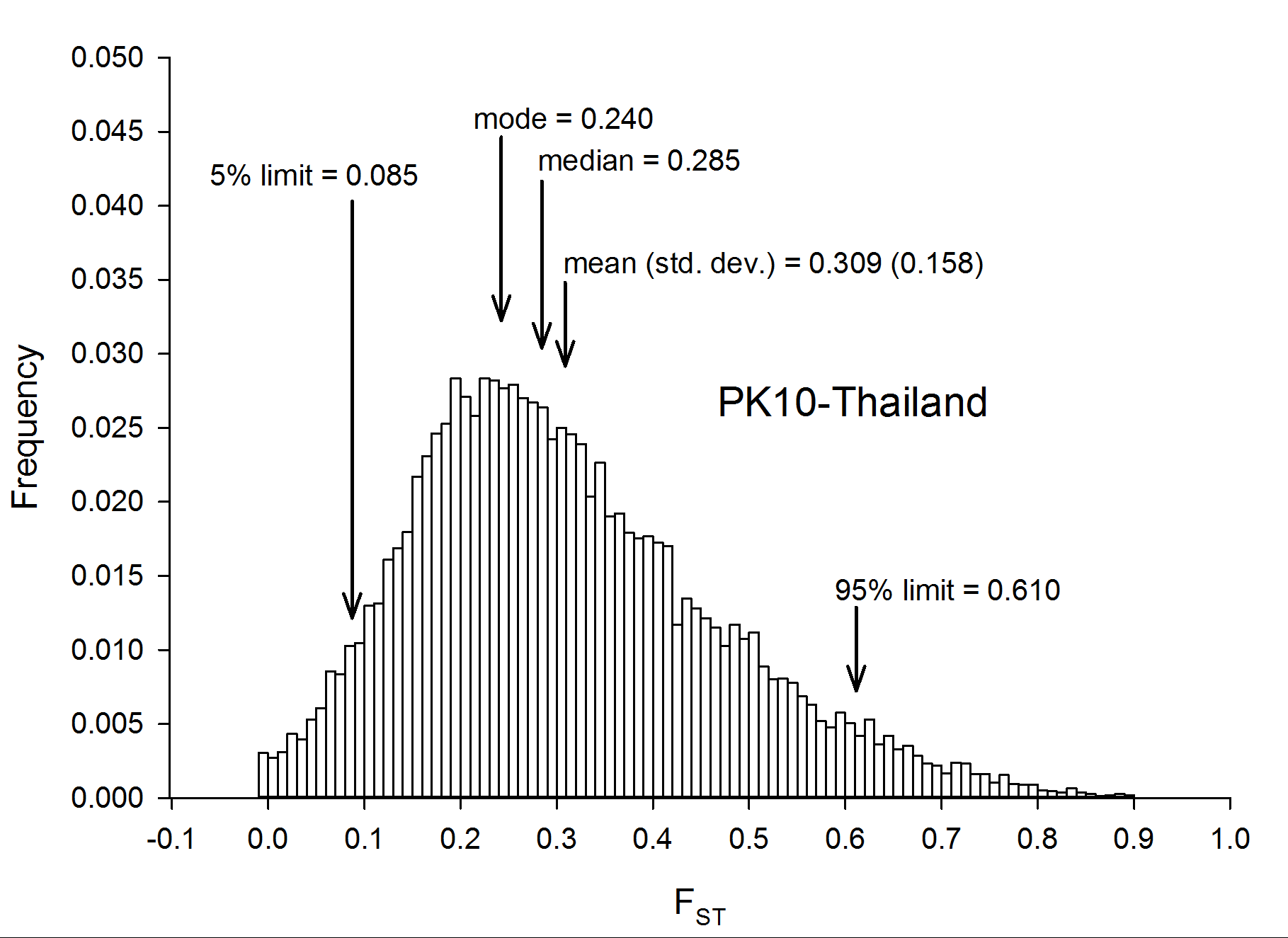

Supplement: Supplementary file 9 [file 571FigureS9.tif]

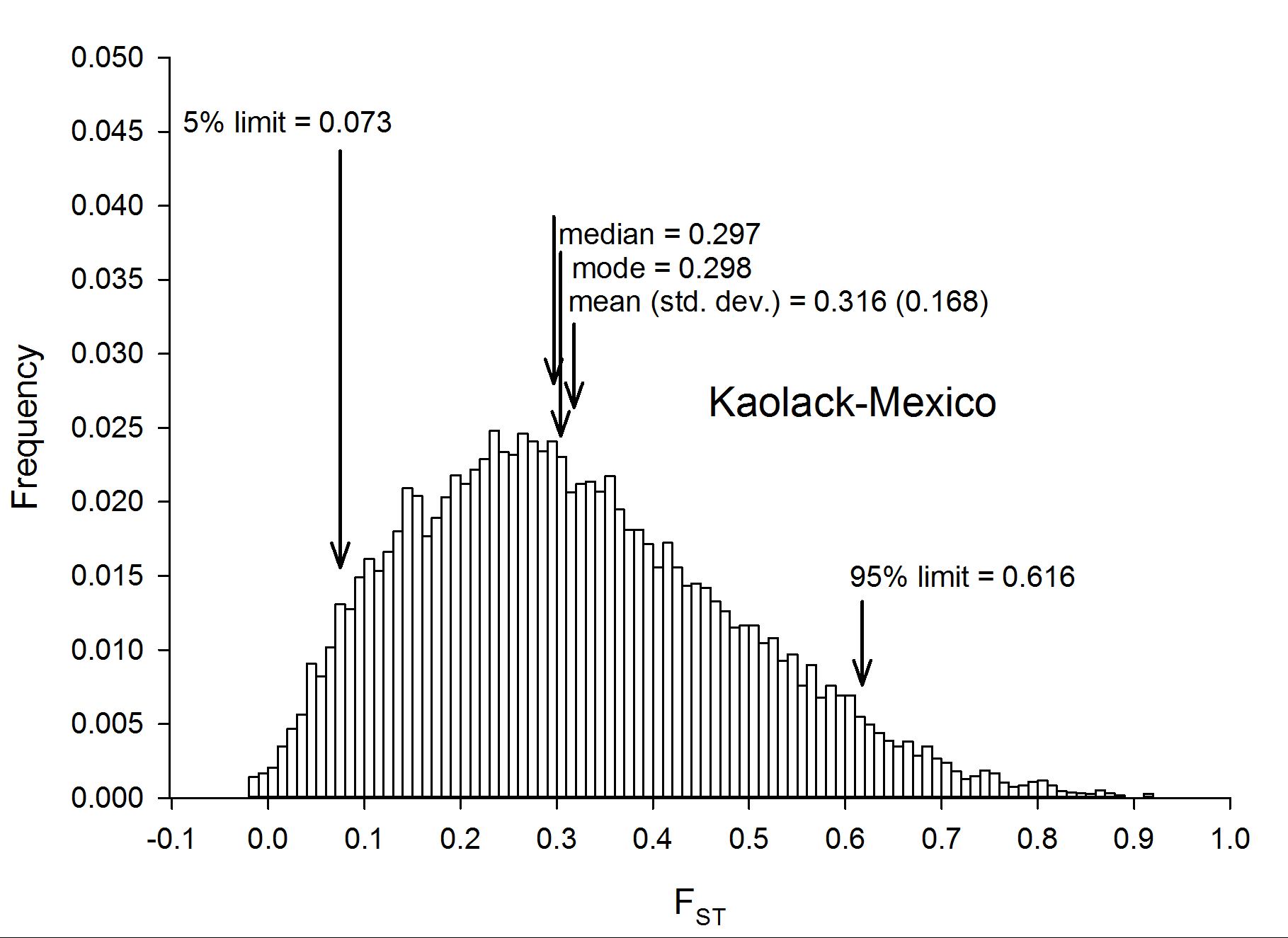

Supplement: Supplementary file 10 [file 571FigureS10.tif]

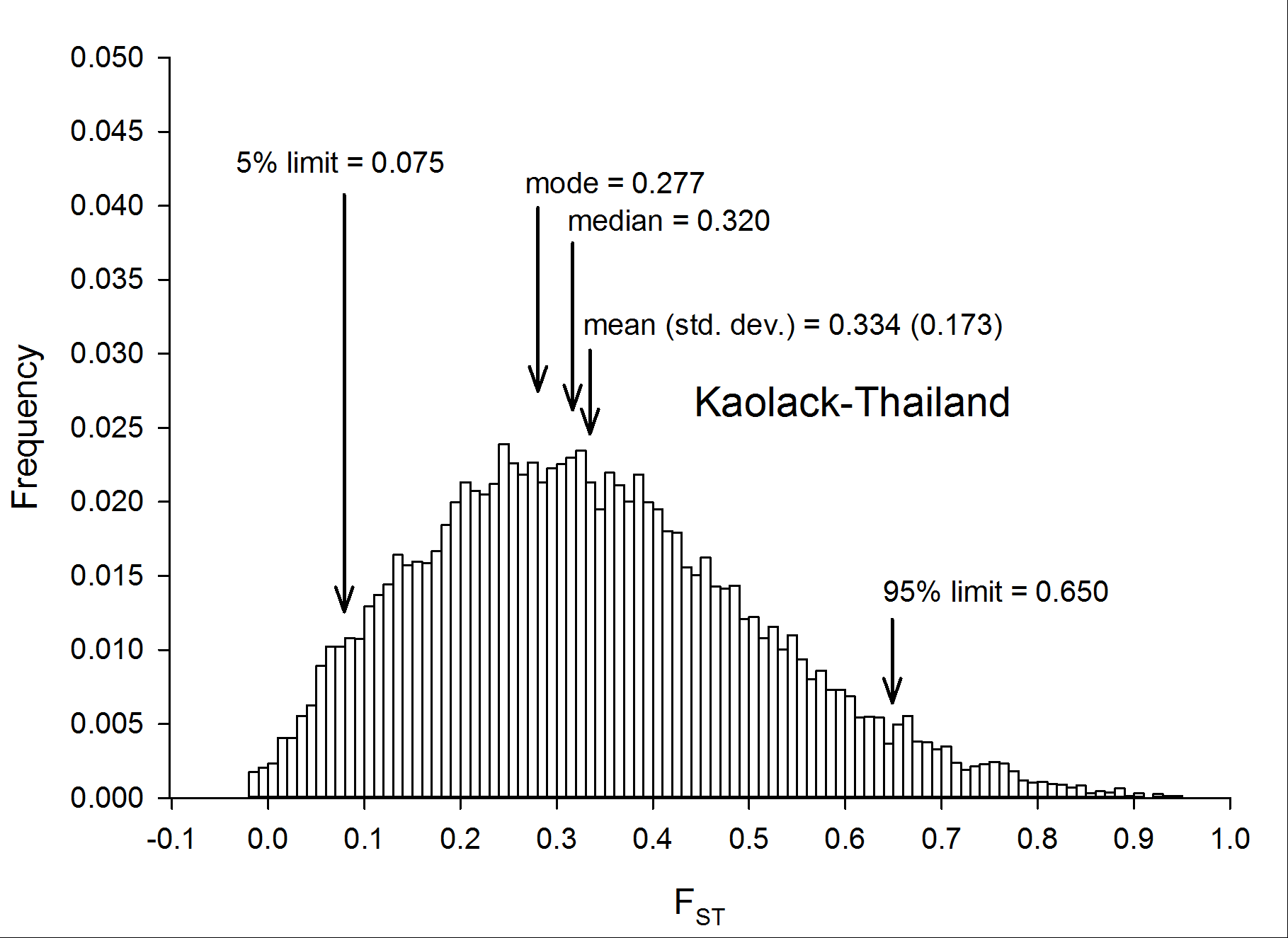

Supplement: Supplementary file 11 [file 571FigureS11.tif]
