# Supplementary material for: Exon-Enriched Libraries Reveal Large Genic Differences Between Aedes aegypti from Senegal, West Africa, and Populations Outside Africa
Source: G3 (Bethesda). 2016 Dec 19;7(2):571–82. doi: 10.1534/g3.116.036053 (PMC5295602; doi:10.1534/g3.116.036053)
Supplement: Supplementary file 12 [file 571TableS1.pdf]

Table S1. Genes in the lower 1% of H_{exp} values.

VectorBase ID	Collection	Hexp	No. SNPs	Function*	Description
AAEL007935-RE	K	0.000	8	CSR	AaGr20a
AAEL017370-RA	K	0.001	361	CSR	odorant receptor
AAEL010277-RA	M	0.001	1611	CSR	AaGr28
AAEL017130-RA	M	0.000	193	CSR	AaGr51
AAEL015499-RA	PKT	0.001,0.001,0.000	548, 476, 479	CSR	5p-1177|odorant-binding ptn 56a
AAEL002626-RA	T	0.001	739	CSR	odorant-binding ptn 56e
AAEL009004-RA	M	0.001	520	CYT/STR	Cuticle ptn
AAEL011444-RA	M	0.001	1047	CYT/STR	pupal cuticle ptn
AAEL006651-RA	P	0.001	3058	CYT/STR	dystrophin
AAEL008999-RA	PT	0.000,0.000	117, 407	CYT/STR	adult cuticle ptn
AAEL000205-RA	K	0.001	479	DIV	hypo. ptn
AAEL000573-RA	K	0.001	323	DIV	grainyhead
AAEL001873-RA	K	0.001	710	DIV	hypo. ptn
AAEL002517-RB	K	0.000	8	DIV	nucleoptn
AAEL002807-RA	K	0.000	45	DIV	hypo. ptn
AAEL002859-RA	K	0.000	42	DIV	hypo. ptn
AAEL003484-RA	K	0.000	30	DIV	zinc finger
AAEL003547-RA	K	0.000	21	DIV	zinc finger
AAEL003672-RA	K	0.001	1497	DIV	zinc finger
AAEL003679-RA	K	0.001	1922	DIV	zinc finger
AAEL004307-RB	K	0.000	41	DIV	n-myc downstream regulated
AAEL004420-RB	K	0.000	3	DIV	nucleoside triphosphate hydrolase
AAEL004835-RA	K	0.000	11	DIV	conserved hypo. ptn
AAEL005502-RA	K	0.000	48	DIV	Kinesin light chain
AAEL005867-RA	K	0.001	1678	DIV	Probable serine/threonine-ptn kinase
AAEL006313-RA	K	0.000	10	DIV	conserved hypo. ptn
AAEL006575-RC	K	0.000	8	DIV	troponin C
AAEL006931-RC	K	0.000	2	DIV	hypo. ptn
AAEL007495-RC	K	0.000	40	DIV	phosphoglycerate mutase
AAEL007913-RA	K	0.000	213	DIV	sulfotransferase (sult)
AAEL008104-RA	K	0.000	16	DIV	fibrinogen and fibronectin
AAEL008263-RA	K	0.001	712	DIV	YY1-associated factor
AAEL008325-RA	K	0.001	189	DIV	hypo. ptn

AAEL008454-RB	K	0.001	943	DIV	Isochorismatase dom-containing
AAEL008834-RB	K	0.000	6	DIV	Tudor dom-containing
AAEL010067-RA	K	0.001	968	DIV	spitz
AAEL010763-RA	K	0.001	574	DIV	hypo. ptn
AAEL011116-RC	K	0.000	14	DIV	14-33 ptn sigma gamma zeta beta/alpha
AAEL011972-RB	K	0.000	40	DIV	actin binding ptn
AAEL012172-RA	K	0.001	1137	DIV	methylthioadenosine phosphorylase
AAEL012188-RA	K	0.001	123	DIV	hypo. ptn
AAEL012476-RA	K	0.001	1134	DIV	RILP-like ptn
AAEL012663-RA	K	0.001	1176	DIV	prolylcarboxypeptidase
AAEL013395-RC	K	0.000	50	DIV	hypo. ptn
AAEL013945-RA	K	0.001	685	DIV	MLX-interacting
AAEL013949-RA	K	0.001	1433	DIV	hypo. ptn
AAEL017253-RD	K	0.000	2	DIV	methionine sulphoxide reductase MsrA
AAEL017462-RC	K	0.000	27	DIV	Hippocampus abundant transcript 1
AAEL017509-RA	KM	0.000,0.000	1, 6	DIV	hypo. ptn
AAEL015302-RA	KMT	0.000,0.001,0.001	78, 643, 605	DIV	Pleckstrin homology dom
AAEL001157-RA	KT	0.001,0.001	1674, 1649	DIV	light ptn
AAEL006926-RA	KT	0.001,0.001	2254, 2202	DIV	hypo. ptn
AAEL009497-RA	KT	0.000,0.000	51, 61	DIV	phosphomannomutase
AAEL011224-RA	KT	0.000,0.000	190, 171	DIV	hypo. ptn
AAEL016985-RA	KT	0.001, 0.001	361, 337	DIV	transmembrane dom
AAEL000319-RA	M	0.000	26	DIV	hypo. ptn
AAEL001064-RA	M	0.000	15	DIV	triacylglycerol lipase
AAEL001577-RA	M	0.000	2	DIV	conserved hypo. ptn
AAEL001966-RA	M	0.000	88	DIV	zinc finger ptn
AAEL002198-RA	M	0.001	287	DIV	oligosaccharyltransferase complex
AAEL002226-RA	M	0.001	1385	DIV	casein kinase
AAEL002232-RA	M	0.001	1428	DIV	Ribonuclease P ptn subunit p30
AAEL002381-RA	M	0.001	259	DIV	High mobility group ptn D
AAEL004071-RA	M	0.001	628	DIV	nitrogen fixation ptn nifu
AAEL004999-RA	M	0.000	3	DIV	hypo. ptn
AAEL005514-RA	M	0.001	557	DIV	hypo. ptn
AAEL005572-RA	M	0.000	28	DIV	hypo. ptn
AAEL005715-RA	M	0.001	778	DIV	Nudix hydrolase dom
AAEL005756-RC	M	0.000	3	DIV	Rhodanese-like dom

AAEL006128-RA	M	0.001	3358	DIV	Meckelin
AAEL006135-RA	M	0.001	1627	DIV	20 Kd nuclear cap binding ptn
AAEL006188-RB	M	0.000	9	DIV	CDC42 small effector ptn
AAEL006359-RA	M	0.000	38	DIV	sulfotransferase (sult)
AAEL006602-RA	M	0.000	13	DIV	hypo. ptn
AAEL007025-RA	M	0.001	1403	DIV	hypo. ptn
AAEL007529-RA	M	0.001	1323	DIV	nucleoptn
AAEL007736-RA	M	0.001	1507	DIV	forkhead ptn/ forkhead ptn dom
AAEL008105-RB	M	0.001	1179	DIV	glutamate cysteine ligase
AAEL009298-RA	M	0.001	1270	DIV	2-Keto-3-deoxy-(6-phospho-)gluconate aldolase
AAEL009361-RA	M	0.000	16	DIV	insect cationic amino acid transporter 2 (iCAT2)
AAEL009452-RA	M	0.000	14	DIV	hypo. ptn
AAEL009627-RA	M	0.000	2	DIV	hypo. ptn
AAEL010128-RA	M	0.000	8	DIV	LRIM4
AAEL010205-RB	M	0.000	35	DIV	conserved hypo. ptn
AAEL010824-RA	M	0.000	2	DIV	seminal fluid ptn
AAEL010901-RB	M	0.000	70	DIV	mannose binding lectin
AAEL011545-RA	M	0.001	380	DIV	cxyorf1
AAEL011546-RA	M	0.000	5	DIV	Vacuolar ATPase assemb integral memb VMA21-like
AAEL011558-RA	M	0.000	65	DIV	metalloptnase
AAEL011578-RA	M	0.001	1748	DIV	serine/threonine ptn kinase
AAEL011606-RA	M	0.000	2	DIV	Transmembrane ptn
AAEL011830-RG	M	0.000	1	DIV	APAF1-interacting
AAEL012896-RA	M	0.000	74	DIV	Zinc finger HIT dom-containing
AAEL013165-RA	M	0.001	409	DIV	Zinc finger
AAEL013276-RA	M	0.000	78	DIV	acid phosphatase
AAEL013574-RB	M	0.000	32	DIV	apolipoptn D
AAEL013596-RA	M	0.001	2003	DIV	phosphatidylinositol 3-kinase regulatory subunit
AAEL013716-RA	M	0.000	1	DIV	Methenyltetrahydrofolate synthetase dom
AAEL013913-RA	M	0.001	335	DIV	hypo. ptn
AAEL014376-RA	M	0.000	6	DIV	hypo. ptn
AAEL014397-RA	M	0.001	207	DIV	grauzone
AAEL014519-RA	M	0.001	721	DIV	cbl-d
AAEL014812-RA	M	0.000	103	DIV	hypo. ptn
AAEL015597-RA	M	0.000	469	DIV	Pleckstrin homology dom

AAEL017033-RA	M	0.001	151	DIV	Disintegrin and metalloptnase dom
AAEL017109-RA	M	0.001	431	DIV	hypo. ptn
AAEL001783-RA	MT	0.000,0.000	7,1	DIV	MSF1 ptn
AAEL002361-RA	MT	0.000,0.000	3, 26	DIV	conserved hypo. ptn
AAEL003521-RA	MT	0.001,0.001	373, 363	DIV	Calcium-transporting ATPase type 2C member 1
AAEL006270-RA	MT	0.000,0.001	1133, 1167	DIV	RAG1-activating ptn
AAEL011951-RB	MT	0.001, 0.001	407, 421	DIV	elongase
AAEL012022-RA	MT	0.000,0.000	72, 5	DIV	hypo. ptn
AAEL000150-RA	P	0.001	993	DIV	nitrilase
AAEL000318-RA	PT	0.000,0.000	112,79	DIV	conserved hypo. ptn
AAEL000475-RB	P	0.000	2	DIV	Beta-catenin-like ptn 1
AAEL000680-RA	P	0.000	4	DIV	hypo. ptn
AAEL001416-RA	P	0.000	15	DIV	Inner nuclear membrane ptn Man1
AAEL001486-RA	P	0.000	61	DIV	Male-specific sperm ptn
AAEL001644-RA	P	0.000	1	DIV	Transmembrane ptn C9orf46
AAEL001679-RA	P	0.000	7	DIV	hypo. ptn
AAEL001822-RA	P	0.000	4	DIV	glucosyl/glucuronosyl transferases
AAEL002060-RA	P	0.000	40	DIV	B box-binding
AAEL002428-RA	P	0.001	94	DIV	BTB/POZ dom-containing
AAEL003323-RA	P	0.000	9	DIV	hypo. ptn
AAEL003348-RC	P	0.000	3	DIV	hypo. ptn
AAEL003376-RA	P	0.000	10	DIV	hypo. ptn
AAEL003880-RA	P	0.001	142	DIV	hypo. ptn
AAEL004351-RB	P	0.000	1	DIV	casein kinase
AAEL006929-RA	P	0.001	3522	DIV	cullin
AAEL007307-RA	P	0.001	2898	DIV	Golgi complex subunit 7
AAEL007407-RA	P	0.000	22	DIV	hypo. ptn
AAEL007640-RA	P	0.000	19	DIV	molybdopterin synthase large subunit
AAEL008057-RA	P	0.001	2374	DIV	myosin light chain kinase
AAEL008379-RC	P	0.000	16	DIV	P38 mapk
AAEL009305-RA	P	0.001	2208	DIV	numb-associated kinase
AAEL009306-RA	P	0.001	1008	DIV	conserved hypo. ptn
AAEL009319-RA	P	0.001	494	DIV	hypo. ptn
AAEL009741-RA	P	0.001	436	DIV	low complexity ptn
AAEL010446-RA	P	0.000	381	DIV	ptn phosphatase 2c
AAEL010726-RA	P	0.000	1	DIV	hypo. ptn

AAEL010802-RA	P	0.000	43	DIV	conserved hypo. ptn
AAEL011053-RA	P	0.000	42	DIV	Zinc finger ptn 337
AAEL011147-RA	P	0.001	819	DIV	Integrator complex subunit 2
AAEL011262-RA	P	0.000	7	DIV	hypo. ptn
AAEL011500-RB	P	0.000	14	DIV	Cdc42 ptn
AAEL011620-RA	P	0.000	4	DIV	hypo. ptn
AAEL011807-RA	P	0.001	1048	DIV	conserved hypo. ptn
AAEL011957-RA	P	0.001	941	DIV	elongase
AAEL012358-RA	P	0.000	8	DIV	hypo. ptn
AAEL012971-RB	P	0.000	1	DIV	Mucin-2
AAEL013248-RB	P	0.000	22	DIV	Cyclin-H
AAEL013376-RA	P	0.001	1112	DIV	conserved hypo. ptn
AAEL013900-RA	P	0.001	2965	DIV	cAMP&cAMP-inhib cgmp 35-cyclic phosphodiesterase
AAEL014287-RA	P	0.000	3	DIV	Deoxycytidylate deaminase
AAEL015099-RI	P	0.000	27	DIV	sumo ligase
AAEL015336-RA	P	0.001	1058	DIV	glutathione-S-transferase dom
AAEL015425-RA	P	0.000	42	DIV	conserved hypo. ptn
AAEL015432-RA	P	0.000	35	DIV	Serine protease easter
AAEL015621-RA	P	0.000	682	DIV	dihydrodipicolinate synthase
AAEL015623-RA	P	0.000	2	DIV	conserved hypo. ptn
AAEL015629-RA	P	0.001	1099	DIV	xaa-pro aminopeptidase
AAEL017102-RA	P	0.001	358	DIV	hypo. ptn
AAEL017142-RA	P	0.000	51	DIV	hypo. ptn
AAEL017170-RA	P	0.000	1	DIV	hypo. ptn
AAEL003793-RA	PK	0.001,0.001	1623, 1642	DIV	hypo. ptn
AAEL006057-RA	PK	0.000,0.000	5,2	DIV	heterogeneous nuclear ribonucleoptn
AAEL007305-RA	PK	0.001,0.001	1574, 1542	DIV	conserved hypo. ptn
AAEL007427-RC	PK	0.000,0.000	33, 122	DIV	zinc finger ptn
AAEL008417-RA	PK	0.001, 0.001	1205, 1124	DIV	Coiled-coil dom-containing
AAEL009651-RA	PK	0.001, 0.001	644, 634	DIV	nascent polypep assoc complex (a subunit(nac alpha)
AAEL011942-RA	PK	0.001, 0.001	948, 771	DIV	zinc finger ptn
AAEL014402-RA	PK	0.000,0.000	4, 14	DIV	grauzone
AAEL015219-RA	PK	0.001, 0.001	469, 535	DIV	HD dom-containing ptn
AAEL015375-RA	PK	0.000,0.000	711, 304	DIV	serine/threonine ptn kinase

AAEL015487-RA	PK	0.001, 0.001	1083, 1085	DIV	zinc finger Noc
AAEL004893-RA	PKM	0.000,0.000,0.000	3, 53, 14	DIV	hypo. ptn
AAEL006965-RA	PKM	0.000,0.000,0.000	9, 38, 11	DIV	hypo. ptn
AAEL008585-RA	PKM	0.001,0.001,0.001	2595, 2560, 2587	DIV	kinase suppressor of ras (ksr)
AAEL011673-RA	PKM	0.001,0.001,0.001	760, 1373, 1353	DIV	cop9 signalosome complex subunit
AAEL008073-RA	PKMT	0.000,0.001,0.001,0.000	1445, 1408, 1466, 1380	DIV	Hyaluronan/mRNA-binding ptn
AAEL014638-RA	PKMT	0.000,0.000,0.000,0.000	58, 36, 93, 56	DIV	hypo. ptn
AAEL014847-RA	PKMT	0.001,0.001,0.001,0.001	1148, 1177, 1152, 1113	DIV	innexin
AAEL000493-RA	PKT	0.001,0.001,0.001	500, 458, 497	DIV	heparan sulphate 6-o-sulfotransferase
AAEL003075-RA	PKT	0.001	2757, 2750, 2678	DIV	Zinc finger
AAEL008588-RA	PKT	0.001,0.001,0.001	1590, 1700, 1492	DIV	innexin
AAEL008722-RA	PKT	0.001,0.001,0.001	3126, 3116, 3130	DIV	zinc finger ptn
AAEL001910-RA	PM	0.000,0.000	53,8	DIV	zinc finger ptn 594
AAEL002664-RA	PM	0.001,0.000	83, 377	DIV	hypo. ptn
AAEL002745-RA	PM	0.000,0.001	71, 289	DIV	nucleoptn
AAEL002973-RA	PM	0.000,0.000	39,34	DIV	zinc finger ptn
AAEL003693-RA	PM	0.000,0.000	74,1	DIV	hypo. ptn
AAEL007300-RA	PM	0.000,0.000	45, 185	DIV	syntaxin binding ptn-123
AAEL012812-RA	PM	0.000,0.000	2, 14	DIV	exosome complex exonuclease RRP41
AAEL002928-RA	PMT	0.001,0.001,0.001	999, 1021, 953	DIV	zinc finger ptn
AAEL011393-RB	PMT	0.001,0.001,0.001	591, 530, 535	DIV	unc-79
AAEL005723-RA	PT	0.001,0.001	1239, 1201	DIV	conserved hypo. ptn
AAEL000042-RA	T	0.000	3	DIV	DnaJ homolog subfamily C member
AAEL000130-RA	T	0.001	864	DIV	nucleoptn dom
AAEL001279-RA	T	0.001	1779	DIV	merozoite surface ptn
AAEL001379-RA	T	0.001	1638	DIV	Coiled-coil dom-containing
AAEL002073-RA	T	0.000	2	DIV	expressed ptn (FTZF1)
AAEL002303-RA	T	0.000	1	DIV	ptn FRA10AC1
AAEL002722-RA	T	0.000	7	DIV	ptn kinase C inhibitor
AAEL003469-RA	T	0.000	38	DIV	NHP2 ptn
AAEL004277-RA	T	0.001	1750	DIV	Metallophosphoesterase dom
AAEL004334-RA	T	0.000	32	DIV	hypo. ptn
AAEL004667-RA	T	0.000	14	DIV	conserved hypo. ptn
AAEL004872-RA	T	0.000	23	DIV	Parkin coregulated gene
AAEL005230-RA	T	0.000	18	DIV	EF-hand dom-containing
AAEL005287-RA	T	0.000	27	DIV	hypo. ptn

AAEL006067-RA	T	0.001	916	DIV	conserved hypo. ptn
AAEL006074-RA	T	0.001	2503	DIV	ER mannosyl-oligosaccharide 12-alpha-mannosidase
AAEL006344-RA	T	0.000	4	DIV	sulfotransferase (sult)
AAEL006545-RA	T	0.001	510	DIV	hypo. ptn
AAEL007020-RA	T	0.001	534	DIV	hypo. ptn
AAEL007161-RA	T	0.001	755	DIV	hypo. ptn
AAEL007265-RA	T	0.000	24	DIV	hypo. ptn
AAEL007427-RA	T	0.001	1160	DIV	zinc finger ptn
AAEL007433-RA	T	0.000	98	DIV	Transmembrane ptn
AAEL007852-RA	T	0.000	7	DIV	hypo. ptn
AAEL008776-RA	T	0.000	218	DIV	Prostaglandin E synthase
AAEL009025-RA	T	0.000	13	DIV	heat shock ptn
AAEL009286-RA	T	0.000	2	DIV	hypo. ptn
AAEL009643-RA	T	0.000	20	DIV	conserved hypo. ptn
AAEL010074-RA	T	0.001	3968	DIV	ptn suppressor of sable
AAEL010705-RA	T	0.001	832	DIV	hypo. ptn
AAEL011667-RA	T	0.000	985	DIV	cir
AAEL012006-RA	T	0.000	60	DIV	E3 ubiquitin-ptn ligase
AAEL012034-RA	T	0.001	1377	DIV	alpha-1 3-mannosyltransferase
AAEL012166-RC	T	0.000	4	DIV	tamozhennic
AAEL012178-RA	T	0.001	2798	DIV	wech
AAEL012280-RA	T	0.001	1100	DIV	peflin ptn w/ long n-terminal hydrophobic dom
AAEL012352-RA	T	0.001	1203	DIV	Prohormone
AAEL012598-RA	T	0.000	8	DIV	hypo. ptn
AAEL013150-RA	T	0.001	1435	DIV	methionine aminopeptidase
AAEL013305-RA	T	0.001	1467	DIV	dihydrofolate reductase-thymidylate synthase
AAEL013408-RA	T	0.000	333	DIV	suppressin
AAEL013465-RA	T	0.000	29	DIV	Inhibof Bruton tyrosine kinase
AAEL013502-RA	T	0.001	864	DIV	ptn painting of fourth
AAEL013580-RA	T	0.000	8	DIV	conserved hypo. ptn
AAEL014510-RA	T	0.001	2047	DIV	sprouty
AAEL014533-RA	T	0.001	4828	DIV	Inhibof Bruton tyrosine kinase
AAEL014819-RA	T	0.001	475	DIV	Hotdog dom
AAEL015045-RA	T	0.000	4	DIV	transcription factor IIIA
AAEL015353-RA	T	0.001	450	DIV	ninjurin a

AAEL015444-RA	T	0.001	754	DIV	conserved hypo. ptn
AAEL017172-RA	T	0.000	12	DIV	Ribokinase-dom
AAEL017471-RA	T	0.000	2	DIV	hypo. ptn
AAEL007765-RB	K	0.001	363	IMM	Serine Protease Inhib(serpin)likely cleavage at K/R
AAEL015533-RA	M	0.001	1291	IMM	kallikrein
AAEL013498-RA	PT	0.001, 0.001	1868, 1960	IMM	Prophenoloxidase subunit A3
AAEL014390-RA	T	0.001	103	IMM	galactose-specific C-type lectin
AAEL015018-RA	T	0.001	512	IMM	toll
AAEL001152-RA	K	0.001	1399	MET	beta-13-galactosyltransferase-6
AAEL010540-RA	K	0.001	2099	MET	alpha-amylase
AAEL001364-RA	M	0.001	1398	MET	glucosyl/glucuronosyl transferases
AAEL003079-RA	P	0.001	1723	MET	glucosyl/glucuronosyl transferases
AAEL008451-RA	PK	0.001, 0.001	1503, 1500	MET	alpha-amylase
AAEL011511-RA	PKT	0.001,0.000,0.001	396, 306, 312	MET	carnitine O-octanoyltransferase
AAEL003076-RA	T	0.001	1854	MET	glucosyl/glucuronosyl transferases
AAEL007323-RA	T	0.001	611	MET	deoxyuridine 5'-triphosphate nucleotidohydrolase
AAEL017833-RA	M	0.000	2	miRNA	miR-92
AAEL017925-RA	PK	0.000,0.000	2, 1	miRNA	miR-190
AAEL017593-RA	T	0.000	2	miRNA	miRNA
AAEL004067-RA	K	0.001	801	MITO	mitochondrial benzodiazepine receptor
AAEL009590-RA	K	0.001	480	MITO	hypo. ptn
AAEL004069-RA	M	0.000	621	MITO	mitochondrial 28S ribosomal ptn S14(s14mt)
AAEL003946-RA	P	0.000	1	MITO	mitochondrial ribosomal ptn S33
AAEL004433-RA	P	0.001	675	MITO	mitochondrial ribosomal ptn L14
AAEL002993-RA	PKM	0.001,0.000,0.000	680, 644, 780	MITO	39S ribosomal ptn L43
AAEL000941-RA	T	0.000	13	MITO	mito inner membrane ptn translocase 8kD-subunit
AAEL009613-RA	T	0.001	542	MITO	mito ribosomal ptn L36
AAEL005860-RA	T	0.000	8	MITO	MIT ATP synthase subunit f
AAEL017711-RA	K	0.000	1	ncRNA	U4 spliceosomal RNA
AAEL017609-RA	M	0.000	223	ncRNA	U1 spliceosomal RNA
AAEL017832-RA	K	0.000	50	ncRNA	Small nucleolar RNA snR54
AAEL017680-RA	K	0.000	81	ncRNA	5.8S rRNA
AAEL017946-RA	K	0.000	6	ncRNA	5.8S rRNA
AAEL017795-RA	KT	0.000,0.000	1, 1	ncRNA	5.8S rRNA

AAEL017756-RA	M	0.000	3	ncRNA	5.8S rRNA
AAEL017789-RA	M	0.000	30	ncRNA	5.8S rRNA
AAEL017867-RA	PK	0.001,0.000	71, 33	ncRNA	5.8S rRNA
AAEL017594-RA	T	0.000	55	ncRNA	5.8S rRNA
AAEL017683-RA	K	0.001	141	ncRNA	5.8S rRNA
AAEL017745-RA	K	0.000	71	ncRNA	28S rRNA
AAEL017856-RA	K	0.000	37	ncRNA	28S rRNA
AAEL017735-RA	KM	0.000,0.001	163, 528	ncRNA	28S rRNA
AAEL017622-RA	M	0.000	25	ncRNA	28S rRNA
AAEL017786-RA	M	0.001	511	ncRNA	28S rRNA
AAEL017585-RA	P	0.001	191	ncRNA	28S rRNA
AAEL017618-RA	PKMT	0.001,0.000,0.000,0.001	105, 236, 112, 227	ncRNA	28S rRNA
AAEL017918-RA	PM	0.000,0.000	1, 300	ncRNA	28S rRNA
AAEL017700-RA	T	0.000	28	ncRNA	28S rRNA
AAEL017909-RA	T	0.001	346	ncRNA	28S rRNA
AAEL017752-RA	K	0.000	66	ncRNA	18s rRNA
AAEL017953-RA	M	0.001	174	ncRNA	18s rRNA
AAEL017614-RA	P	0.001	234	ncRNA	18s rRNA
AAEL017915-RA	P	0.001	484	ncRNA	18s rRNA
AAEL017944-RA	P	0.001	509	ncRNA	18s rRNA
AAEL017780-RA	PK	0.000,0.001	179, 139	ncRNA	18s rRNA
AAEL017686-RA	PMT	0.000,0.000,0.000	97, 97, 97	ncRNA	18s rRNA
AAEL017782-RA	T	0.000	61	ncRNA	18s rRNA
AAEL000002-RA	K	0.000	54	PROT	hypo. ptn
AAEL002121-RA	K	0.001	857	PROT	26S proteasome complex subunit
AAEL004173-RA	K	0.001	1550	PROT	26S proteasome non-ATPase regulatory subunit
AAEL008600-RA	K	0.001	1497	PROT	zinc carboxypeptidase
AAEL003921-RA	KM	0.000,0.000	11,8	PROT	anopheles stephensi ubiquitin
AAEL006414-RA	M	0.001	876	PROT	trypsin
AAEL014642-RA	MT	0.001, 0.001	526, 499	PROT	ubiquitin conjugating enzyme
AAEL009689-RA	P	0.001	1162	PROT	serine protease
AAEL013890-RA	P	0.000	41	PROT	26S proteasome non-ATPase regulatory subunit
AAEL017574-RA	P	0.001	307	PROT	Trypsin-1
AAEL006902-RA	PK	0.000,0.000	4, 6	PROT	serine-type enodpeptidase
AAEL002273-RA	T	0.000	6	PROT	trypsin
AAEL006960-RA	T	0.001	2122	PROT	calpain

AAEL008781-RA	T	0.000	83	PROT	serine-type enodpeptidase
AAEL014568-RA	T	0.000	8	PROT	Peptidase S1 dom
AAEL002835-RA	K	0.001	2727	ReDox	Oxidative stress-induced
AAEL010075-RA	K	0.001	1049	ReDox	oxidoreductase
AAEL010146-RA	K	0.001	1522	ReDox	3-hydroxyacyl-coa dehyrogenase
AAEL011158-RB	K	0.001	982	ReDox	disulfide oxidoreductase
AAEL015628-RA	KM	0.000, 0.001	1310, 865	ReDox	glycine dehydrogenase
AAEL014208-RA	KMT	0.001,0.001,0.001	2004, 2028, 1997	ReDox	cytochrome P450 CYP307B1
AAEL003011-RA	M	0.001	294	ReDox	NADH dehydrogenase
AAEL006081-RA	M	0.000	1	ReDox	NADPH fad oxidoreductase
AAEL014684-RA	M	0.000	16	ReDox	CYP6F3
AAEL017165-RA	M	0.001	2003	ReDox	Cytochrome P450 4V3
AAEL015002-RA	MT	0.000,0.000	9, 10	ReDox	aldo-keto reductase
AAEL007385-RB	P	0.000	26	ReDox	ferritin subunit 1
AAEL009685-RA	P	0.001	1253	ReDox	oxidoreductase
AAEL012614-RA	P	0.000	77	ReDox	NADP-specific isocitrate dehydrogenase
AAEL015625-RA	P	0.000	260	ReDox	NAD+ dep glycerol-3-phosphate dehydrogenase
AAEL015630-RA	P	0.000	957	ReDox	pyrroline-5-carboxylate dehydrogenase
AAEL008072-RA	PT	0.001,0.001	767, 773	ReDox	NADH-plastoquinone oxidoreductase
AAEL005796-RB	K	0.000	37	R/T/T	eukaryotic translation initiation factor 4e
AAEL013761-RB	K	0.000	42	R/T/T	ADP-ribosylation factor arf
AAEL014498-RA	K	0.001	814	R/T/T	Exosome complex exonuclease
AAEL017030-RA	KM	0.001, 0.001	798, 758	R/T/T	RNA-binding ptn Rsf1
AAEL000513-RA	KMT	0.000,0.000,0.001	133, 95, 133	R/T/T	histone H4
AAEL015449-RA	KMT	0.001,0.001,0.001	276, 358, 98	R/T/T	ribosome biogenesis ptn brix
AAEL003851-RA	KT	0.001,0.000	80,69	R/T/T	histone H2A
AAEL003866-RA	KT	0.000,0.001	17,71	R/T/T	histone H4
AAEL003382-RA	M	0.000	10	R/T/T	Ro ribonucleoptn autoantigen
AAEL005245-RA	M	0.001	3742	R/T/T	DNA repair ptn rad50
AAEL010025-RA	M	0.001	973	R/T/T	SAGA-associated factor
AAEL014423-RA	M	0.001	2167	R/T/T	Mitotic spindle assembly checkpoint ptnMAD1
AAEL014831-RA	M	0.001	506	R/T/T	RNA polymerase II subunit Rpb10
AAEL015645-RA	M	0.001	870	R/T/T	RNA binding motif ptn
AAEL015677-RA	M	0.000	128	R/T/T	histone H2B
AAEL000432-RA	P	0.001	1110	R/T/T	nucleosome assembly ptn
AAEL002435-RD	P	0.000	28	R/T/T	Broad-complex core ptn isoform 6

AAEL012171-RA	P	0.001	2409	R/T/T	RNA recognition motif ptn split ends
AAEL015626-RA	P	0.001	254	R/T/T	Ribosomal ptn L11 methyltransferase
AAEL015060-RA	PK	0.001, 0.001	1122, 1095	R/T/T	Rad51A ptn
AAEL009071-RA	PKM	0.001,0.001,0.001	305, 659, 413	R/T/T	60S ribosomal ptn L7
AAEL008637-RA	PKT	0.001,0.001,0.001	918, 934, 819	R/T/T	cdk5
AAEL005196-RA	PM	0.001,0.001	812, 863	R/T/T	PDZ dom
AAEL006141-RA	PM	0.001,0.001	509, 659	R/T/T	40S ribosomal ptn S3a
AAEL015006-RA	PMT	0.000,0.000,0.000	163, 194, 300	R/T/T	60S ribosomal ptn L23
AAEL010218-RA	PT	0.000,0.000	25, 28	R/T/T	GATA transcription factor (GATAa)
AAEL010180-RA	T	0.001	1071	R/T/T	conserved hypo. ptn
AAEL010463-RA	T	0.001	595	R/T/T	Homeobox ptn rough
AAEL011999-RA	T	0.001	448	R/T/T	histone H4
AAEL014748-RA	T	0.001	3363	R/T/T	DNA repair ptn rad50
AAEL007766-RA	K	0.001	839	SigT	ras GTPase
AAEL003378-RA	M	0.001	1575	SigT	GPCR Orphan/ Class A Family
AAEL015236-RA	M	0.001	445	SigT	signal recognition particle 9kD-subunit
AAEL011970-RA	P	0.001	749	SigT	GPCR Purine/Adenosine Family
AAEL015250-RA	P	0.001	676	SigT	AmphiWnt7b
AAEL011621-RA	PT	0.000,0.000	53, 34	SigT	C-Type Lectin (CTL) -mannose binding
AAEL013645-RA	T	0.001	1179	SigT	FMRFamide-dom
AAEL017126-RA	T	0.001	258	SigT	hypo. ptn
AAEL002105-RA	K	0.001	1092	SPLICE	conserved hypo. ptn
AAEL008228-RA	MT	0.001, 0.001	608, 614	SPLICE	small nuclear ribonucleoptn
AAEL016080-RA	K	0.000	3	tRNA	Pseudogene Ala(AGC antisense)
AAEL016122-RA	K	0.000	7	tRNA	Arg (TCT antisense)
AAEL016266-RA	K	0.000	11	tRNA	Ala pseudogene
AAEL016313-RA	K	0.000	8	tRNA	Glu(TTC antisense)
AAEL016542-RA	K	0.000	3	tRNA	Phe(GAA antisense)
AAEL016703-RA	K	0.000	1	tRNA	Gln(CTG antisense)
AAEL016709-RA	K	0.000	5	tRNA	Ala(AGC antisense)
AAEL016784-RA	K	0.000	5	tRNA	Ala(AGC antisense)
AAEL016897-RA	K	0.000	4	tRNA	Pseudogene Ala(AGC antisense)
AAEL016918-RA	K	0.000	5	tRNA	Thr(TGT antisense)
AAEL016935-RA	K	0.000	1	tRNA	Asp(GTC antisense)
AAEL016940-RA	K	0.000	12	tRNA	Asp(GTC antisense)
AAEL016065-RA	M	0.000	9	tRNA	Ser(CGA antisense)

AAEL016121-RA	M	0.000	10	tRNA	Glu(CTC antisense)
AAEL016632-RA	M	0.000	3	tRNA	His(GTG antisense)
AAEL016943-RA	M	0.000	5	tRNA	Pro(AGG antisense)
AAEL016094-RA	P	0.000	2	tRNA	Pseudogene Ala(AGC antisense)
AAEL016907-RA	P	0.000	1	tRNA	Pseudogene Ala(AGC antisense)
AAEL016906-RA	PMT	0.000,0.000,0.000	1, 1, 1	tRNA	Glu pseudogene
AAEL016022-RA	T	0.000	9	tRNA	Lys(CTT antisense)
AAEL016089-RA	T	0.000	19	tRNA	Ala pseudogene
AAEL016115-RA	T	0.000	44	tRNA	Lys(CTT antisense)
AAEL006019-RD	K	0.000	10	TRP	voltage-gated sodium channel
AAEL006582-RB	K	0.001	84	TRP	calcium-transporting ATPase sarcoplasmic/ER type
AAEL012960-RA	K	0.001	2354	TRP	importin alpha
AAEL015538-RA	K	0.001	928	TRP	microtubule-associated ptn tau
AAEL014223-RA	KM	0.000,0.000	87, 118	TRP	vitellogenin receptor (VgR)
AAEL000524-RA	KT	0.001,0.001	309, 315	TRP	regulating synaptic membrane exocytosis ptn
AAEL008470-RA	M	0.001	477	TRP	hypo. ptn
AAEL017576-RA	M	0.001	911	TRP	Calcium-transporting ATPase sarcoplasmic/ER type
AAEL004019-RA	MT	0.001,0.001	721, 731	TRP	Acetylcholine receptor subunit beta-like 2
AAEL011432-RA	P	0.001	4085	TRP	plexin b
AAEL012478-RA	PK	0.001, 0.001	1302, 1296	TRP	glucose transporter
AAEL013146-RA	PK	0.000,0.001	188, 174	TRP	mfs transporter
AAEL012198-RA	PMT	0.001,0.001,0.001	917, 814, 870	TRP	ADP ATP carrier ptn
AAEL007288-RB	PT	0.001,0.001	366, 321	TRP	dynamin
AAEL000930-RC	T	0.001	314	TRP	Transmembrane ptn
AAEL002761-RG	T	0.000	22	TRP	tropomyosin invertebrate
AAEL009636-RA	T	0.000	6	TRP	Exportin-7
AAEL009813-RA	T	0.001	3443	TRP	glutamate receptor 7 (ampa)
AAEL013033-RA	T	0.001	374	TRP	hypo. ptn
AAEL014643-RA	T	0.001	645	TRP	UDP-sugar transporter UST74c (fringe connection ptn)
AAEL000211-RA	K	0.001	596	UNK	conserved hypo. ptn
AAEL000841-RA	K	0.000	42	UNK	hypo. ptn
AAEL003557-RA	K	0.001	895	UNK	Lebercilin-like
AAEL004665-RA	K	0.000	379	UNK	Ninjurin dom
AAEL008414-RA	K	0.001	4186	UNK	hypo. ptn

AAEL009433-RA	K	0.001	645	UNK	conserved hypo. ptn
AAEL013618-RA	K	0.001	2130	UNK	conserved hypo. ptn
AAEL013857-RA	K	0.001	125	UNK	hypo. ptn
AAEL013946-RA	K	0.001	781	UNK	hypo. ptn
AAEL000183-RA	KM	0.001, 0.001	325, 470	UNK	hypo. ptn
AAEL003016-RA	KM	0.001,0.001	1994, 2087	UNK	hypo. ptn
AAEL010400-RA	KM	0.000,0.000	753, 800	UNK	hypo. ptn
AAEL015121-RA	KMT	0.001,0.001,0.000	470, 455, 453	UNK	hypo. ptn
AAEL017057-RA	KMT	0.000,0.001,0.001	668, 663, 668	UNK	hypo. ptn
AAEL017759-RA	KMT	0.001,0.000,0.000	141, 56, 8	UNK	unknown
AAEL017047-RA	KT	0.001, 0.001	331, 314	UNK	hypo. ptn
AAEL001259-RA	M	0.000	306	UNK	hypo. ptn
AAEL003007-RA	M	0.001	634	UNK	hypo. ptn
AAEL006134-RA	M	0.000	488	UNK	Coiled-coil dom
AAEL006275-RA	M	0.001	1173	UNK	conserved hypo. ptn
AAEL007086-RA	M	0.001	425	UNK	hypo. ptn
AAEL007321-RA	M	0.001	1073	UNK	LysM and peptidoglycan-binding dom
AAEL007703-RA	M	0.000	8	UNK	conserved hypo. ptn
AAEL008778-RA	M	0.001	1130	UNK	conserved hypo. ptn
AAEL011286-RA	M	0.000	30	UNK	conserved hypo. ptn
AAEL012038-RA	M	0.001	2086	UNK	hypo. ptn
AAEL013167-RA	M	0.000	255	UNK	conserved hypo. ptn
AAEL017108-RA	M	0.001	332	UNK	hypo. ptn
AAEL017274-RA	M	0.001	346	UNK	hypo. ptn
AAEL017550-RA	M	0.001	339	UNK	hypo. ptn
AAEL004591-RB	MT	0.000,0.000	8, 59	UNK	hypo. ptn
AAEL008066-RA	MT	0.001,0.001	622, 625	UNK	hypo. ptn
AAEL016994-RA	MT	0.000, 0.001	478, 384	UNK	hypo. ptn
AAEL018158-RA	MT	0.001, 0.001	942, 879	UNK	Zinc finger
AAEL003760-RA	P	0.001	755	UNK	conserved hypo. ptn
AAEL005193-RA	P	0.001	823	UNK	hypo. ptn
AAEL008065-RA	P	0.001	1032	UNK	Myosin light chain kinase-like
AAEL009315-RA	P	0.001	458	UNK	hypo. ptn
AAEL009827-RA	P	0.001	891	UNK	hypo. ptn
AAEL013865-RA	P	0.001	461	UNK	conserved hypo. ptn
AAEL014661-RA	P	0.001	429	UNK	hypo. ptn

AAEL015376-RA	P	0.001	345	UNK	hypo. ptn
AAEL017024-RA	P	0.001	219	UNK	hypo. ptn
AAEL017791-RA	P	0.000	8	UNK	unknown
AAEL017853-RA	P	0.001	151	UNK	unknown
AAEL018303-RA	P	0.001	936	UNK	Lebercilin-like
AAEL001753-RA	PK	0.001,0.001	758, 585	UNK	hypo. ptn
AAEL004446-RA	PK	0.001,0.001	1568, 1554	UNK	hypo. ptn
AAEL005713-RA	PK	0.001,0.001	670, 674	UNK	Cyclin-dependent kinase 2-associated ptn
AAEL009821-RA	PK	0.001, 0.001	3051, 3067	UNK	hypo. ptn
AAEL013298-RA	PK	0.001,0.000	795, 516	UNK	serine protease
AAEL015473-RA	PK	0.001,0.000	304, 212	UNK	zinc finger
AAEL017894-RA	PK	0.001,0.000	158, 180	UNK	unknown
AAEL007163-RA	PKMT	0.000,0.001,0.000,0.001	1465, 1494, 1409,1446	UNK	hypo. ptn
AAEL015521-RA	PKT	0.001,0.000,0.000	385, 353, 390	UNK	Phosphofurin acidic cluster sorting-dom
AAEL017176-RA	PKT	0.001,0.001,0.001	336, 317, 330	UNK	hypo. ptn
AAEL015537-RA	PM	0.001, 0.001	649, 421	UNK	BTB-POZ-like
AAEL017413-RA	PM	0.001, 0.001	393, 419	UNK	hypo. ptn
AAEL017551-RA	PMT	0.001,0.001,0.001	241, 245, 146	UNK	hypo. ptn
AAEL014442-RA	PT	0.001, 0.001	596, 525	UNK	hypo. ptn
AAEL001773-RA	T	0.000	120	UNK	conserved hypo. ptn
AAEL004123-RA	T	0.001	690	UNK	hypo. ptn
AAEL004937-RA	T	0.001	684	UNK	hypo. ptn
AAEL005503-RA	T	0.001	925	UNK	Actin cytoskeleton-regulatory dom
AAEL006238-RA	T	0.001	394	UNK	conserved hypo. ptn
AAEL007053-RB	T	0.001	199	UNK	hypo. ptn
AAEL007620-RA	T	0.000	117	UNK	hypo. ptn
AAEL009683-RA	T	0.001	483	UNK	hypo. ptn
AAEL011397-RA	T	0.001	1146	UNK	hypo. ptn
AAEL013011-RA	T	0.001	389	UNK	hypo. ptn
AAEL013646-RA	T	0.001	862	UNK	conserved hypo. ptn
AAEL017485-RA	T	0.001	336	UNK	hypo. ptn
AAEL017540-RA	T	0.000	159	UNK	hypo. ptn

* Functional classes: ‘CSR’, chemosensory reception; ‘CYT/STR’, cytoskeletal and structural; ‘DIV’, diverse functions; ‘IMM’, immunity-and apoptosis-related; ‘MET’, metabolism; ’miRNA’ microRNA; ‘MITO’, mitochondrial; ‘ncRNA’ non-coding RNA’; ‘PROT’, proteolysis or 26S proteasome function; ‘R/T/T’, replication, transcription, and translation; ‘ReDox’, reduction/oxidation and oxidoreductive stress; ‘SigT’, Signal

Transduction; 'SPLICE', RNA processing or pre-processing and splicing activity or regulation; ‘TRP’, transport or signaling; ‘UNK’, unknown functions.
