# Supplementary material for: Exon-Enriched Libraries Reveal Large Genic Differences Between Aedes aegypti from Senegal, West Africa, and Populations Outside Africa
Source: G3 (Bethesda). 2016 Dec 19;7(2):571–82. doi: 10.1534/g3.116.036053 (PMC5295602; doi:10.1534/g3.116.036053)
Supplement: Supplementary file 13 [file 571TableS2.pdf]

Table S2. Genes in the upper 1% of H_{exp} values.

VectorBase ID	Collection	Hexp	No. SNPs	Function*	Description
AAEL009800-RA	P	0.061	512	CYT/STR	cuticle prot
AAEL000029-RA	T	0.048	2083	DIV	dopachrome-conversion enzyme (DCE)
AAEL000076-RA	K	0.196	2	DIV	dimethyladenosine transferase
AAEL000475-RB	KT	0.073,0.082	179,68	DIV	Beta-catenin-like
AAEL000560-RA	M	0.067	38	DIV	hypo prot
AAEL000810-RA	KMT	0.069,0.05,0.049	10611,8578,7884	DIV	prot kinase c
AAEL001838-RA	P	0.058	66	DIV	Prefoldin subunit
AAEL002264-RA	KT	0.086,0.074	147,32	DIV	conserved hypo prot
AAEL004011-RA	M	0.051	9	DIV	hypo prot
AAEL004164-RA	K	0.111	437	DIV	conserved hypo prot
AAEL004351-RB	K	0.103	118	DIV	casein kinase
AAEL004351-RC	K	0.09	95	DIV	casein kinase
AAEL004372-RA	PKMT	0.127,0.16,0.126,0.057	1044,1188,1118,1020	DIV	Lipid storage droplets surface-binding
AAEL004689-RB	PK	0.091,0.083	51,68	DIV	RING finger domain
AAEL004872-RA	M	0.132	61	DIV	Parkin coregulated
AAEL004985-RA	K	0.113	58	DIV	house keepingprot
AAEL005073-RA	M	0.051	3292	DIV	Golgin subfamily A member 6-like
AAEL005551-RA	PKMT	0.102,0.096,0.064,0.09	1137,1204,1281,1132	DIV	Trichohyalin
AAEL005756-RC	T	0.069	67	DIV	Rhodanese-like domain
AAEL005877-RC	K	0.081	16	DIV	Activator of 90 kDa heat shock prot ATPase homolog
AAEL005977-RA	KM	0.069,0.08	833,241	DIV	chondroitin 4-sulfotransferase
AAEL006341-RA	PK	0.064,0.082	72,588	DIV	conserved hypo prot
AAEL006353-RA	K	0.081	58	DIV	sulfotransferase (sult)
AAEL006445-RA	T	0.048	1545	DIV	GPI-anchored wall transfer prot 1
AAEL006575-RC	P	0.063	8	DIV	troponin C
AAEL006600-RC	T	0.054	241	DIV	juvenile hormone-inducible prot
AAEL006632-RB	KM	0.078,0.08	67,34	DIV	Receptor expression-enhancing prot
AAEL006820-RA	PKMT	0.069,0.085,0.059,0.06	1577,1618,1570,1553	DIV	lipid storage droplets surface binding prot 2 (lsd2)
AAEL006996-RA	M	0.088	59	DIV	conserved hypo prot
AAEL007294-RA	T	0.046	2477	DIV	Dromyosuppressin
AAEL007614-RA	T	0.12	4	DIV	conserved hypo prot
AAEL007820-RB	K	0.086	156	DIV	LSM12 homolog A
AAEL007953-RA	T	0.076	559	DIV	SPRY domain

AAEL008012-RA	PKT	0.114,0.109,0.124	59,132,1	DIV	hypo prot
AAEL008058-RB	K	0.107	18	DIV	liprin a (lar-interacting prot a synapse defective prot 2)
AAEL008092-RA	K	0.072	157	DIV	hypo prot
AAEL008114-RA	PK	0.071,0.093	981,998	DIV	p15-2b prot
AAEL008200-RA	P	0.065	155	DIV	conserved hypo prot
AAEL008532-RA	T	0.066	22	DIV	NULL
AAEL008556-RB	PKMT	0.117,0.105,0.088,0.093	83,120,88,78	DIV	prp4
AAEL008900-RA	T	0.046	214	DIV	p15-2a prot
AAEL009052-RA	PKM	0.079,0.095,0.158	100,229,19	DIV	conserved hypo prot
AAEL009212-RE	K	0.07	12	DIV	lola
AAEL009219-RA	P	0.058	4630	DIV	predicted prot
AAEL009997-RA	PMT	0.065,0.056,0.061	969,933,952	DIV	hypo prot
AAEL010170-RB	K	0.092	106	DIV	ras-related prot Rab-8A
AAEL010172-RA	PKM	0.076,0.088,0.081	145,221,64	DIV	Leucokinins
AAEL010194-RA	PK	0.058,0.085	105,65	DIV	hypo prot
AAEL010281-RA	MT	0.075,0.06	102,120	DIV	Zinc finger
AAEL010576-RE	K	0.097	12	DIV	modifier of mdg4
AAEL010897-RA	MT	0.052,0.053	1391,1111	DIV	SH3 domain-containing
AAEL011083-RB	PKMT	0.105,0.1,0.055,0.112	164,219,48,107	DIV	NULL
AAEL011098-RA	PKMT	0.078,0.086,0.061,0.054	2907,3073,2830,2620	DIV	Nucleoside diphosphate kinase
AAEL011208-RA	PMT	0.067,0.064,0.055	1358,1590,1305	DIV	Av71 muscle cell intermediate filament
AAEL011414-RC	PT	0.058,0.046	119,5	DIV	high mobility group non-histone prot
AAEL012119-RA	PKMT	0.096,0.072,0.096,0.099	609,1285,309,257	DIV	Nucleolin
AAEL012231-RA	K	0.145	71	DIV	hypo prot
AAEL012576-RA	K	0.07	55	DIV	pyruvate kinase
AAEL012612-RB	K	0.127	36	DIV	serine/threonine prot kinase
AAEL012887-RA	MT	0.086,0.077	47,3	DIV	conserved hypo prot
AAEL012971-RA	PKMT	0.079,0.068,0.051,0.057	325,427,378,350	DIV	Mucin-2
AAEL012971-RB	K	0.082	22	DIV	Mucin-2
AAEL013038-RA	M	0.058	1571	DIV	Diacylglycerol kinase
AAEL013203-RB	PK	0.139,0.102	5,127	DIV	Ubiquitin carboxyl-terminal hydrolase
AAEL013464-RA	T	0.059	208	DIV	WD repeat-containing
AAEL013574-RB	PK	0.071,0.072	145,190	DIV	apolipoprot D
AAEL013681-RA	M	0.063	19	DIV	hypo prot
AAEL013877-RG	PKMT	0.06,0.069,0.059,0.051	1115,1604,598,366	DIV	glucosamine-6-phosphate isomerase
AAEL014039-RA	K	0.072	74	DIV	conserved hypo prot

AAEL014668-RA	PKMT	0.086,0.091,0.08,0.083	3397,3409,3092,3211	DIV	ankyrin 23/unc44
AAEL014741-RA	PKM	0.082,0.094,0.061	1915,1736,1358	DIV	serine/threonine-prot kinase ripk4
AAEL014742-RA	PKMT	0.135,0.139,0.067,0.061	3063,2875,2562,2721	DIV	ankyrin 23/unc44
AAEL014956-RA	MT	0.051,0.053	1983,1924	DIV	internalin A
AAEL015143-RA	P	0.074	37	DIV	glycine rich RNA binding prot
AAEL015454-RA	M	0.075	82	DIV	conserved hypo prot
AAEL017027-RA	T	0.076	6	DIV	hypo prot
AAEL017039-RA	T	0.128	26	DIV	Alcohol dehydrogenase
AAEL017112-RA	T	0.053	65	DIV	hypo prot
AAEL017469-RA	MT	0.074,0.052	64,16	DIV	hypo prot
AAEL017506-RA	PKMT	0.073,0.077,0.124,0.06	172,251,20,61	DIV	hypo prot
AAEL003139-RB	M	0.057	116	FAT	short-chain dehydrogenase
AAEL009231-RA	PT	0.059,0.051	1268,1244	FAT	Lipid storage droplet-1
AAEL006212-RA	T	0.047	2116	IMM	toll-like receptor
AAEL014141-RB	PKMT	0.08,0.069,0.085,0.084	124,131,116,82	IMM	Serine Protease Inhibitor (serpin)likely cleavage at S/M
AAEL005520-RB	PMT	0.062,0.068,0.056	182,77,121	MET	carbonic anhydrase
AAEL012113-RA	T	0.054	331	MET	vacuolar ATP synthase proteolipid subunit
AAEL013132-RB	K	0.077	38	MET	6-phosphofructo-2-kinase/fructose-2 6-bisphosphatase
AAEL017670-RA	PMT	0.064,0.061,0.054	249,223,212	miRNA	miRNA
AAEL000526-RA	T	0.047	28	MITO	ATP synthase subunit f mito
AAEL000619-RA	PKM	0.067,0.103,0.057	724,726,658	MITO	ATP synthase subunit f mito
AAEL001718-RA	PKMT	0.071,0.084,0.054,0.06	1223,1256,1161,1172	MITO	ATP synthase subunit f mito
AAEL001885-RA	PMT	0.059,0.058,0.061	53,159,73	MITO	ATP synthase subunit f mito
AAEL002431-RA	MT	0.057,0.058	494,549	MITO	ATP synthase subunit f mito
AAEL002897-RA	PKMT	0.068,0.081,0.053,0.048	742,749,729,747	MITO	ATP synthase subunit f mito
AAEL006000-RC	MT	0.056,0.054	227,176	MITO	cytochrome c oxidase polypeptide 7A1
AAEL006118-RA	MT	0.056,0.057	107,61	MITO	mito ribosomal prot L55
AAEL008059-RA	KMT	0.068,0.057,0.049	971,789,815	MITO	ATP synthase subunit f mito
AAEL008582-RA	P	0.086	23	MITO	30S ribosomal prot S2(prokaryotic and organellar)
AAEL009589-RA	M	0.059	189	MITO	Endonuclease G mito
AAEL011010-RA	KT	0.106,0.05	725,710	MITO	ATP synthase subunit f mito
AAEL011110-RA	MT	0.051,0.049	471,460	MITO	ATP synthase subunit f mito
AAEL011391-RB	K	0.12	26	MITO	mito ribosomal prot S9
AAEL014532-RA	T	0.058	529	MITO	ATP synthase subunit f mito
AAEL014681-RA	PKT	0.065,0.093,0.049	970,960,909	MITO	ATP synthase subunit f mito
AAEL017508-RA	M	0.057	80	MITO	mito dicarboxylate carrier

AAEL017637-RA	K	0.099	19	ncRNA	U6 spliceosomal RNA
AAEL017645-RA	PKMT	0.079,0.086,0.084,0.077	125,161,157,58	ncRNA	U2 spliceosomal RNA
AAEL017739-RA	PKM	0.089,0.105,0.127	126,111,31	ncRNA	U5 spliceosomal RNA
AAEL017763-RA	P	0.127	20	ncRNA	U6 spliceosomal RNA
AAEL017814-RA	K	0.073	127	ncRNA	U1 spliceosomal RNA
AAEL017860-RA	K	0.109	110	ncRNA	U1 spliceosomal RNA
AAEL017888-RA	PKM	0.102,0.088,0.074	108,115,109	ncRNA	U5 spliceosomal RNA
AAEL017921-RA	T	0.059	190	ncRNA	5.8S ribosomal RNA
AAEL003877-RA	MT	0.056,0.049	3170,3250	PROT	ubiquitin
AAEL004121-RB	K	0.109	97	PROT	ubiquitin-conjugating enzyme E2 q
AAEL009898-RA	M	0.05	1933	PROT	proteasome subunit alpha type
AAEL014007-RA	K	0.1	26	PROT	Serine protease easter
AAEL017399-RA	T	0.048	3378	PROT	Glutamyl aminopeptidase
AAEL006588-RA	PKMT	0.069,0.111,0.069,0.063	840,994,727,654	ReDox	conserved hypo prot
AAEL007463-RA	K	0.077	59	ReDox	5 10-methylenetetrahydrofolate reductase
AAEL007523-RA	PMT	0.065,0.069,0.072	7423,7203,7165	ReDox	peroxisomal n1-acetyl-spermine/spermidine oxidase
AAEL009685-RB	PMT	0.082,0.081,0.077	392,254,213	ReDox	oxidoreductase
AAEL013007-RA	M	0.056	695	ReDox	cytochrome c oxidase subunit VB
AAEL015588-RA	M	0.058	12	ReDox	5p-1106|hydroxyacyl dehydrogenase
AAEL001484-RA	PMT	0.082,0.076,0.079	628,567,310	R/T/T	chromosome region maintenance prot 1/exportin
AAEL002368-RA	PM	0.079,0.078	113,113	R/T/T	40S ribosomal prot S27
AAEL003382-RA	K	0.089	227	R/T/T	Ro ribonucleoprot autoantigen
AAEL004830-RA	PKMT	0.079,0.074,0.085,0.089	509,597,494,374	R/T/T	60S ribosomal prot L18a
AAEL006785-RB	PKT	0.104,0.073,0.093	127,105,74	R/T/T	60S ribosomal prot L18a
AAEL007048-RB	PKT	0.112,0.102,0.089	151,163,80	R/T/T	Zinc finger
AAEL007925-RA	T	0.047	743	R/T/T	histone H2A
AAEL008026-RA	T	0.048	370	R/T/T	MADF domain
AAEL008447-RA	T	0.053	717	R/T/T	60S ribosomal prot L18a
AAEL009118-RA	PKM	0.099,0.101,0.207	122,160,3	R/T/T	histone H2A
AAEL009400-RC	PM	0.08,0.087	213,148	R/T/T	ccr4-associated factor
AAEL010335-RA	T	0.046	257	R/T/T	60S ribosomal prot L18a
AAEL010468-RB	PMT	0.068,0.079,0.073	281,173,138	R/T/T	DNA-directed RNA polymerase I 12kD-subunit
AAEL010550-RA	PMT	0.069,0.066,0.069	778,775,700	R/T/T	60S ribosomal prot L18
AAEL011089-RB	K	0.112	107	R/T/T	ribonucleoprot
AAEL011251-RA	M	0.058	35	R/T/T	RNA binding motif prot
AAEL011286-RA	P	0.061	46	R/T/T	conserved hypo prot

AAEL012618-RA	K	0.069	253	R/T/T	RNA-binding
AAEL012921-RA	PMT	0.066,0.062,0.063	440,460,471	R/T/T	ribosomal prot L36
AAEL013682-RA	M	0.058	1101	R/T/T	zinc finger prot
AAEL016019-RA	PKMT	0.107,0.081,0.057,0.105	104,83,61,59	R/T/T	tRNA-Asp Asp ATC
AAEL017516-RC	K	0.072	39	R/T/T	60S ribosomal prot L23a
AAEL004246-RB	PK	0.1,0.126	39,36	SigT	still life sif
AAEL006091-RA	PK	0.07,0.094	40,112	SigT	rab6
AAEL006786-RC	PKM	0.07,0.078,0.099	58,456,96	SigT	GTPase rho
AAEL017480-RA	PKMT	0.125,0.147,0.103,0.092	2005,1954,1988,2005	SigT	Ankyrin-1
AAEL016262-RA	PK	0.137,0.102	44,118	Trna	Ala (not confirmed)
AAEL016369-RA	PK	0.059,0.083	71,155	tRNA	Ala (not confirmed)
AAEL016478-RA	PK	0.061,0.07	85,189	tRNA	Ala (not confirmed)
AAEL016754-RA	K	0.073	140	tRNA	Ala (not confirmed)
AAEL016890-RA	P	0.062	61	tRNA	Ala (not confirmed)
AAEL016048-RA	K	0.124	53	tRNA	Ala(AGC antisense)
AAEL016379-RA	PKMT	0.074,0.08,0.078,0.077	237,241,180,184	tRNA	Ala(AGC antisense)
AAEL016076-RA	K	0.11	46	tRNA	Ala(AGC antisense)
AAEL016250-RA	PKM	0.075,0.089,0.117	46,98,9	tRNA	Ala(AGC antisense)
AAEL016283-RA	PKM	0.065,0.076,0.078	119,156,107	tRNA	Ala(AGC antisense)
AAEL016300-RA	P	0.231	1	tRNA	Ala(AGC antisense)
AAEL016322-RA	M	0.052	101	tRNA	Ala(AGC antisense)
AAEL016323-RA	K	0.08	120	tRNA	Ala(AGC antisense)
AAEL016335-RA	PKT	0.105,0.085,0.095	23,24,37	tRNA	Ala(AGC antisense)
AAEL016368-RA	PKM	0.106,0.113,0.074	117,130,38	tRNA	Ala(AGC antisense)
AAEL016376-RA	PMT	0.059,0.052,0.06	222,138,123	tRNA	Ala(AGC antisense)
AAEL016405-RA	P	0.066	208	tRNA	Ala(AGC antisense)
AAEL016411-RA	PKMT	0.089,0.073,0.267,0.066	90,111,3,18	tRNA	Ala(AGC antisense)
AAEL016421-RA	K	0.116	37	tRNA	Ala(AGC antisense)
AAEL016425-RA	PKMT	0.071,0.071,0.076,0.094	147,120,41,42	tRNA	Ala(AGC antisense)
AAEL016427-RA	PK	0.099,0.103	91,179	tRNA	Ala(AGC antisense)
AAEL016433-RA	P	0.058	6	tRNA	Ala(AGC antisense)
AAEL016606-RA	T	0.048	105	tRNA	Ala(AGC antisense)
AAEL016623-RA	PK	0.095,0.116	59,52	tRNA	Ala(AGC antisense)
AAEL016650-RA	PKM	0.072,0.07,0.091	141,201,81	tRNA	Ala(AGC antisense)
AAEL016672-RA	PKMT	0.071,0.08,0.066,0.069	207,205,153,173	tRNA	Ala(AGC antisense)
AAEL016735-RA	P	0.067	92	tRNA	Ala(AGC antisense)

AAEL016760-RA	PK	0.1,0.086	70,133	tRNA	Ala(AGC antisense)
AAEL016761-RA	P	0.061	23	tRNA	Ala(AGC antisense)
AAEL016782-RA	PKMT	0.079,0.072,0.078,0.077	156,163,144,82	tRNA	Ala(AGC antisense)
AAEL016827-RA	PKM	0.133,0.118,0.11	83,148,64	tRNA	Ala(AGC antisense)
AAEL016937-RA	PKM	0.098,0.11,0.056	96,175,28	tRNA	Ala(AGC antisense)
AAEL016950-RA	P	0.087	21	tRNA	Ala(AGC antisense)
AAEL016829-RA	T	0.071	58	tRNA	Ala(AGC antisense)
AAEL016248-RA	T	0.058	30	tRNA	Ala(AGC antisense)
AAEL016801-RA	P	0.124	1	tRNA	Ala(AGC antisense)
AAEL016917-RA	P	0.065	26	tRNA	Ala(AGC antisense)
AAEL016075-RA	PKMT	0.087,0.078,0.071,0.081	182,186,134,126	tRNA	Ala(AGC antisense)
AAEL016160-RA	PKM	0.065,0.08,0.072	118,114,62	tRNA	Ala(AGC antisense)
AAEL016052-RA	M	0.056	67	tRNA	Ala(AGC antisense)
AAEL016601-RA	PKMT	0.074,0.077,0.087,0.07	120,147,98,87	tRNA	Ala(TGC antisense)
AAEL016536-RA	K	0.069	90	tRNA	Arg(ACG antisense)
AAEL016554-RA	P	0.059	19	tRNA	Arg(ACG antisense)
AAEL016592-RA	M	0.05	33	tRNA	Arg(ACG antisense)
AAEL016256-RA	PKMT	0.096,0.093,0.088,0.089	288,459,196,215	tRNA	Arg(TCG antisense)
AAEL016667-RA	PKMT	0.084,0.091,0.089,0.079	160,165,160,144	tRNA	Asn(GTT antisense)
AAEL016231-RA	PMT	0.06,0.058,0.069	144,158,142	tRNA	Asn(GTT antisense)
AAEL016444-RA	K	0.114	21	tRNA	Asp(GTC antisense)
AAEL016541-RA	P	0.09	34	tRNA	Asp(GTC antisense)
AAEL016955-RA	T	0.046	203	tRNA	Asp(GTC antisense)
AAEL016202-RA	T	0.046	228	tRNA	Asp(GTC antisense)
AAEL016400-RA	MT	0.053,0.053	205,172	tRNA	Cys(GCA antisense)
AAEL016492-RA	PK	0.062,0.082	39,18	tRNA	Cys(GCA antisense)
AAEL016145-RA	M	0.053	33	tRNA	Glu (not confirmed)
AAEL016392-RA	P	0.064	45	tRNA	Glu (not confirmed)
AAEL016132-RA	PK	0.099,0.121	43,87	tRNA	Glu(CTC antisense)
AAEL016148-RA	KM	0.091,0.079	56,45	tRNA	Glu(CTC antisense)
AAEL016473-RA	M	0.082	26	tRNA	Glu(CTC antisense)
AAEL016557-RA	PK	0.072,0.09	56,126	tRNA	Glu(TTC antisense)
AAEL016856-RA	PK	0.069,0.085	54,97	tRNA	Glu(TTC antisense)
AAEL016705-RA	M	0.055	7	tRNA	Gly (not confirmed)
AAEL016508-RA	M	0.062	34	tRNA	Gly (not confirmed)
AAEL016191-RA	PMT	0.061,0.074,0.059	13,22,8	tRNA	Gly(GCC antisense)

AAEL016009-RA	PKMT	0.085,0.08,0.089,0.081	41,159,8,58	tRNA	Gly(GCC antisense)
AAEL016274-RA	M	0.054	25	tRNA	Gly(TCC antisense)
AAEL016689-RA	P	0.062	16	tRNA	Gly(TCC antisense)
AAEL016879-RA	PMT	0.069,0.065,0.065	417,334,386	tRNA	His(GTG antisense)
AAEL016458-RA	PK	0.062,0.068	83,135	tRNA	His(GTG antisense)
AAEL016104-RA	K	0.121	58	tRNA	Leu(CAG antisense)
AAEL016396-RA	T	0.062	86	tRNA	Leu(AAG antisense)
AAEL016436-RA	P	0.11	5	tRNA	Leu(GAG antisense)
AAEL016803-RA	PKMT	0.11,0.087,0.095,0.099	183,295,116,147	tRNA	Leu(GAG antisense)
AAEL016406-RA	T	0.06	14	tRNA	Leu(TAG antisense)
AAEL016865-RA	M	0.06	55	tRNA	Leu(TAG antisense)
AAEL016239-RA	PKMT	0.073,0.083,0.117,0.073	136,149,10,65	tRNA	Lys(CTT antisense)
AAEL016268-RA	T	0.057	29	tRNA	Lys(CTT antisense)
AAEL016178-RA	PK	0.073,0.068	12,53	tRNA	Lys(CTT antisense)
AAEL016669-RA	P	0.062	8	tRNA	Met(CAT antisense)
AAEL016226-RA	K	0.072	51	tRNA	Met(CAT antisense)
AAEL016943-RA	PT	0.061,0.072	129,82	tRNA	Pro(AGG antisense)
AAEL016520-RA	PKT	0.062,0.096,0.12	28,92,15	tRNA	Pro(TGG antisense)
AAEL016291-RA	T	0.116	2	tRNA	Pseudogene Ala(AGC antisense)
AAEL016304-RA	K	0.084	41	tRNA	Pseudogene Ala(AGC antisense)
AAEL016314-RA	K	0.135	29	tRNA	Pseudogene Ala(AGC antisense)
AAEL016364-RA	PKMT	0.105,0.116,0.108,0.103	150,180,119,46	tRNA	Pseudogene Ala(AGC antisense)
AAEL016378-RA	PK	0.09,0.158	13,95	tRNA	Pseudogene Ala(AGC antisense)
AAEL016418-RA	PKM	0.075,0.07,0.093	118,89,87	tRNA	Pseudogene Ala(AGC antisense)
AAEL016502-RA	K	0.114	86	tRNA	Pseudogene Ala(AGC antisense)
AAEL016572-RA	PKM	0.087,0.095,0.098	127,221,32	tRNA	Pseudogene Ala(AGC antisense)
AAEL016748-RA	PKMT	0.112,0.082,0.115,0.099	97,206,55,45	tRNA	Pseudogene Ala(AGC antisense)
AAEL016795-RA	P	0.069	34	tRNA	Pseudogene Ala(AGC antisense)
AAEL016798-RA	MT	0.053,0.056	117,65	tRNA	Pseudogene Ala(AGC antisense)
AAEL016804-RA	PKMT	0.108,0.132,0.13,0.13	108,111,66,84	tRNA	Pseudogene Ala(AGC antisense)
AAEL016821-RA	K	0.109	31	tRNA	Pseudogene Ala(AGC antisense)
AAEL016846-RA	K	0.117	64	tRNA	Pseudogene Ala(AGC antisense)
AAEL016916-RA	PKMT	0.092,0.092,0.098,0.076	179,276,91,163	tRNA	Pseudogene Ala(AGC antisense)
AAEL016089-RA	K	0.108	186	tRNA	Pseudogene Ala(AGC antisense)
AAEL016390-RA	PKMT	0.086,0.083,0.087,0.072	301,333,61,69	tRNA	Pseudogene Glu(CTC antisense)
AAEL016832-RA	T	0.048	64	tRNA	Pseudogene Phe(AAA antisense)

AAEL016219-RA	PKMT	0.102,0.098,0.076,0.116	107,146,119,62	tRNA	Pseudogene Val(GAC antisense)
AAEL016067-RA	PMT	0.06,0.059,0.049	85,65,65	tRNA	Ser(CGA antisense))
AAEL016223-RA	PKMT	0.077,0.074,0.082,0.054	170,195,148,128	tRNA	Ser(AGA antisense)
AAEL016065-RA	P	0.063	118	tRNA	Ser(CGA antisense)
AAEL016066-RA	PKMT	0.068,0.067,0.07,0.068	431,496,392,431	tRNA	Ser(CGA antisense)
AAEL016951-RA	PK	0.06,0.068	35,165	tRNA	Ser(not confirmed)
AAEL016180-RA	P	0.073	51	tRNA	Thr(AGT antisense)
AAEL016237-RA	K	0.096	47	tRNA	Tyr(GTA antisense)
AAEL016150-RA	KM	0.083,0.059	137,6	tRNA	Tyr(GTA antisense)
AAEL016154-RA	PK	0.084,0.116	48,122	tRNA	Tyr(GTA antisense)
AAEL016157-RA	KT	0.069,0.062	111,54	tRNA	Tyr(GTA antisense)
AAEL016316-RA	PKM	0.102,0.079,0.231	146,83,1	tRNA	Val(AAC antisense)
AAEL016627-RA	P	0.12	29	tRNA	Val(AAC antisense)
AAEL016880-RA	PMT	0.065,0.067,0.064	186,181,145	tRNA	Val(AAC antisense)
AAEL016043-RA	K	0.092	5	tRNA	Val(AAC antisense)
AAEL016404-RA	PKM	0.083,0.087,0.092	174,198,74	tRNA	Val(not confirmed)
AAEL000309-RB	M	0.064	50	TRP	Cytokine receptor
AAEL001125-RA	PKMT	0.091,0.132,0.056,0.046	600,511,572,616	TRP	p15-2b prot
AAEL001516-RA	PK	0.071,0.081	10,138	TRP	5p-1810|vesicle associated prot
AAEL002436-RC	PKT	0.095,0.091,0.085	77,70,22	TRP	Trimeric intracellular cation channel type B
AAEL004268-RA	PMT	0.059,0.06,0.056	2671,2060,1723	TRP	Sialin Sodium/sialic acid cotransporter
AAEL007521-RB	K	0.126	8	TRP	importin beta-2
AAEL007580-RA	PK	0.079,0.092	1030,1107	TRP	NTF2-related export prot
AAEL010579-RA	K	0.071	1498	TRP	Lipid storage droplets surface-binding
AAEL011825-RC	K	0.071	11	TRP	aryl hydrocarbon receptor
AAEL012288-RA	M	0.059	803	TRP	sugar transporter
AAEL009600-RB	PKMT	0.078,0.072,0.078,0.087	731,904,446,329	TRP	20-hydroxy-ecdysone receptor
AAEL000304-RC	MT	0.058,0.056	202,107	UNK	conserved hypo prot
AAEL000348-RA	PMT	0.076,0.069,0.075	148,168,117	UNK	conserved hypo prot
AAEL000473-RA	PMT	0.059,0.051,0.053	2242,2130,1876	UNK	hypo prot
AAEL001495-RB	M	0.057	673	UNK	conserved hypo prot
AAEL001561-RB	K	0.091	52	UNK	conserved hypo prot
AAEL001712-RA	PM	0.064,0.053	340,247	UNK	hypo prot
AAEL002180-RA	PMT	0.066,0.059,0.05	777,793,769	UNK	hypo prot
AAEL002573-RA	T	0.05	285	UNK	hypo prot
AAEL002604-RA	PT	0.059,0.055	1194,1375	UNK	SH3 domain-containing

AAEL002884-RA	MT	0.05,0.051	858,787	UNK	hypo prot
AAEL005161-RA	T	0.046	838	UNK	hypo prot
AAEL005758-RA	MT	0.051,0.048	497,502	UNK	conserved hypo prot
AAEL006470-RA	PKMT	0.071,0.07,0.064,0.055	3511,5905,2863,2873	UNK	hypo prot
AAEL006644-RA	T	0.047	3351	UNK	hypo prot
AAEL006846-RA	M	0.058	292	UNK	hypo prot
AAEL006914-RA	PMT	0.063,0.062,0.078	419,201,187	UNK	hypo prot
AAEL006943-RA	MT	0.057,0.056	9491,8900	UNK	hypo prot
AAEL007571-RA	M	0.054	862	UNK	hypo prot
AAEL007645-RA	MT	0.053,0.051	590,546	UNK	hypo prot
AAEL007734-RA	T	0.047	311	UNK	conserved hypo prot
AAEL008808-RA	T	0.054	526	UNK	hypo prot
AAEL009065-RA	T	0.05	815	UNK	hypo prot
AAEL009322-RA	M	0.052	293	UNK	hypo prot
AAEL009552-RA	PMT	0.059,0.055,0.047	712,773,799	UNK	hypo prot
AAEL009633-RA	T	0.049	811	UNK	conserved hypo prot
AAEL010325-RA	PT	0.059,0.054	894,1058	UNK	hypo prot
AAEL010634-RA	PKMT	0.066,0.069,0.078,0.098	106,133,68,57	UNK	hypo prot
AAEL010690-RA	M	0.055	349	UNK	hypo prot
AAEL010746-RA	PMT	0.066,0.059,0.057	1354,1287,1170	UNK	hypo prot
AAEL010849-RA	PKT	0.06,0.091,0.046	1619,1913,1253	UNK	NTF2-related
AAEL011160-RA	MT	0.054,0.05	452,453	UNK	predicted prot
AAEL011207-RA	KT	0.068,0.048	2854,1711	UNK	hypo prot
AAEL011618-RA	M	0.058	237	UNK	conserved hypo prot
AAEL011829-RA	T	0.049	250	UNK	hypo prot
AAEL011894-RA	T	0.076	84	UNK	hypo prot
AAEL011971-RA	KM	0.07,0.055	1141,1053	UNK	Nicastrin
AAEL012196-RA	M	0.057	751	UNK	conserved hypo prot
AAEL012331-RA	M	0.053	452	UNK	SH3 domain-containing
AAEL012849-RB	PKT	0.078,0.085,0.067	81,82,57	UNK	hypo prot
AAEL013655-RA	PMT	0.058,0.055,0.058	1108,811,713	UNK	conserved hypo prot
AAEL013838-RA	PKMT	0.063,0.079,0.059,0.063	1835,2092,1371,907	UNK	hypo prot
AAEL014726-RA	MT	0.05,0.055	2205,1751	UNK	conserved hypo prot
AAEL014955-RA	PMT	0.06,0.06,0.064	3322,1980,1599	UNK	tartan prot
AAEL015091-RA	PMT	0.064,0.071,0.092	1341,759,322	UNK	hypo prot
AAEL015509-RA	T	0.05	219	UNK	conserved hypo prot

AAEL016980-RA	MT	0.054,0.056	908,1016	UNK	hypo prot
AAEL017279-RA	T	0.047	436	UNK	hypo prot
AAEL017605-RA	T	0.056	87	UNK	hypo prot
AAEL008230-RA	KMT	0.094,0.096,0.097	466,183,131	UNK	hypo prot

* Functional classes: ‘CYT/STR’, cytoskeletal and structural; ‘DIV’, diverse functions; FAT, Fatty Acid Metabolism; ‘IMM’, immunity-and apoptosis-related; ‘MET’, metabolism; ’miRNA’ microRNA; ‘MITO’, mitochondrial; ‘ncRNA’ non-coding RNA’; ‘PROT’, proteolysis or 26S proteasome function; ‘R/T/T’, replication, transcription, and translation; ‘ReDox’, reduction/oxidation and oxidoreductive stress; ‘SigT’, Signal Transduction; ‘TRP’, transport or signaling; ‘UNK’, unknown functions.
