# Supplementary material for: Exon-Enriched Libraries Reveal Large Genic Differences Between Aedes aegypti from Senegal, West Africa, and Populations Outside Africa
Source: G3 (Bethesda). 2016 Dec 19;7(2):571–82. doi: 10.1534/g3.116.036053 (PMC5295602; doi:10.1534/g3.116.036053)
Supplement: Supplementary file 14 [file 571TableS3.pdf]

Table S3. Comparisons yielding lower 1% of FST values.

VectorBase	FST PK10 Mer	nSN Ps	FST PK10 Kao	nSNPs	FST PK10 Thai	nSN Ps	FST Kao Mer	nSN Ps	FST Kao Thai	nSN Ps	FST Mer Thai	nS NPs	Group	Annotation
AAEL000158-RA	0.013	1145	0.008	1625	0.016	1045	-	-	-	-	-	-	DIV	T-box transcription factor tbx2
AAEL002419-RA	-	-	0.007	2418	-	-	0.011	1634	0.010	1454	-	-	DIV	sarcolemmal associated ptn-2
AAEL004700-RA	0.016	721	0.008	1007	0.009	665	-	-	-	-	-	-	DIV	cdkl1/4
AAEL005500-RA	0.017	1503	0.004	2410	0.015	1473	-	-	-	-	-	-	DIV	cons hypo ptn
AAEL009372-RA	0.017	142	0.003	296	0.005	79	-	-	-	-	-	-	DIV	Meiotically up-regulated gene
AAEL009458-RB	0.012	544	0.004	767	0.007	530	0.010	543	0.008	502	-	-	DIV	syntaxin 4
AAEL011236-RA	0.017	615	0.009	1132	0.007	473	-	-	-	-	-	-	DIV	Calcineurin-binding
AAEL015064-RB	0.016	385	0.008	562	0.013	286	0.014	368	0.010	264	-	-	DIV	Small ubiquitin-related
AAEL017184-RA	0.008	390	0.007	879	0.013	465	-	-	-	-	-	-	DIV	Kelch-like
AAEL007765-RC	0.007	403	0.005	572	0.011	317	0.006	395	0.007	293	-	-	IMM	Serpin likely cleavage at K/R. Transcript A
AAEL007765-RD	0.016	323	0.005	572	0.011	317	-	-	-	-	-	-	IMM	Serpin likely cleavage at K/R. Transcript A
AAEL007308-RA	0.012	946	0.009	1500	0.009	923	0.016	897	0.011	873	-	-	MET	Alpha-1 2-mannosyltransferase ALG9
AAEL017730-RA	0.001	133	0.001	160	0.006	141	0.006	130	0.002	135	0.005	111	miRNA	microRNA
AAEL006000-RC	0.009	185	0.002	194	0.008	139	0.004	185	0.002	139	0.005	130	MITO	cytochrome c oxidase polypeptide 7A1
AAEL017921-RA	0.009	152	0.005	175	0.006	137	-	-	-	-	-	-	ncRNA	5.8S ribosomal RNA
AAEL007316-RA	0.008	7866	0.004	10178	0.006	7401	0.007	7663	0.005	7195	-	-	PROT	Kyphoscoliosis peptidase
AAEL009685-RA	-	-	-	-	0.008	135	-	-	0.012	131	0.002	138	ReDox	oxidoreductase
AAEL007505-RA	0.010	2268	0.008	2909	0.008	2266	-	-	-	-	-	-	R/T/T	iroquois-class homeodomain ptn irx

AAEL008026-RA	0.004	277	0.000	389	0.009	291	0.004	263	0.007	280	0.005	257	R/T/T	MADF domain
AAEL008575-RA	0.014	2254	0.008	3205	0.009	1914	-	-	-	-	-	-	R/T/T	regulator of chromosome condensation
AAEL010468-RB	0.010	72	0.004	126	0.011	66	0.016	71	0.011	62	-	-	R/T/T	DNA-directed RNA polymerase I 12kD-subu
AAEL011162-RA	0.013	721	0.007	1137	0.015	602	-	-	-	-	-	-	R/T/T	Anaphase Promoting Complex putative
AAEL000737-RB	0.016	267	-	-	-	-	0.008	239	-	-	0.002	276	SigT	rab6 GTPase activating ptn gapcena (rabga
AAEL006936-RA	0.011	1099	0.006	1750	0.013	916	-	-	-	-	-	-	SigT	cons hypo ptn
AAEL015250-RA	-	-	0.005	17	-	-	0.002	15	0.000	27	-	-	SigT	AmphiWnt7b putative
AAEL016379-RA	0.003	143	0.003	200	0.007	141	-	-	-	-	-	-	tRNA	Ala (AGC antisense)
AAEL016479-RA	0.014	100	0.007	130	0.015	117	-	-	-	-	-	-	tRNA	Ala (AGC antisense)
AAEL016672-RA	0.011	139	0.004	169	0.009	139	-	-	0.007	135	0.006	128	tRNA	Ala (AGC antisense)
AAEL016256-RA	0.001	177	0.001	212	0.003	169	-	-	-	-	-	-	tRNA	Arg (TCG antisense)
AAEL016667-RA	0.002	143	-	-	0.005	130	0.003	143	0.001	129	0.005	126	tRNA	Asn (GTT antisense)
AAEL016955-RA	0.011	135	0.004	220	0.012	114	0.012	128	0.013	104			tRNA	Asp (GTC antisense)
AAEL016842-RA	0.016	7	0.007	18	0.004	10	-	-	-	-	-	-	tRNA	Pro (AGG antisense)
AAEL016401-RA	0.017	35	-	-	-	-	0.012	58	-	-	0.004	32	tRNA	Pro (AGG antisense)
AAEL016066-RA	0.014	272	0.001	316	0.006	317	-	-	-	-	-	-	tRNA	Ser (CGA antisense)
AAEL002472-RA	0.006	1032	0.004	1092	0.003	1083	0.010	973	0.006	1011	-	-	UNK	hypo ptn
AAEL003948-RA	0.016	460	0.005	512	0.016	525	-	-	-	-	-	-	UNK	hypo ptn
AAEL004208-RA	0.007	267	-	-	0.005	311	0.010	222	0.006	273	0.002	224	UNK	hypo ptn
AAEL006284-RA	0.010	61	0.004	73	0.010	72	0.013	49	0.007	60			UNK	hypo ptn
AAEL006470-RA	0.012	1449	0.006	2062	0.015	1356	-	-	-	-	-	-	UNK	hypo ptn
AAEL006914-RA	0.010	145	0.001	209	0.012	155	-	-	-	-	-	-	UNK	hypo ptn
AAEL006943-RA	0.006	5823	0.004	6455	0.005	5656	0.010	5470	0.005	5401	0.002	515	UNK	hypo ptn

												1		
AAEL007314-RA	0.011	2006	0.004	2672	0.007	1944	0.008	1925	0.006	1863	-	-	UNK	hypo ptn
AAEL007319-RA	0.015	263	-	-	-	-	0.012	244	-	-	0.004	230	UNK	cons hypo ptn
AAEL008230-RA	0.009	146	0.006	150	0.004	80	0.009	157	0.009	79	-	-	UNK	hypo ptn
AAEL009095-RA	0.010	347	-	-	0.010	340	0.010	345	0.012	356	0.005	292	UNK	hypo ptn
AAEL009818-RA	0.009	1412	0.003	1966	0.008	1058	0.013	1375	0.007	1008	-	-	UNK	cons hypo ptn
AAEL010049-RA	0.009	1022	0.008	1327	0.014	963	-	-	-	-	-	-	UNK	hypo ptn
AAEL013402-RB	0.015	751	0.007	410	0.012	131	-	-	-	-	-	-	UNK	hypo ptn
AAEL014954-RA	0.017	517	0.006	1239	0.006	423	0.012	498	0.007	410	-	-	UNK	hypo ptn
AAEL016980-RA	-	-	-	-	0.014	394	-	-	0.009	387	0.006	337	UNK	hypo ptn

* Functional classes: ‘DIV’, diverse functions; ‘IMM’, immunity-and apoptosis-related; ‘MET’, metabolism; ’miRNA’ microRNA; ‘MITO’, mitochondrial; ‘ncRNA’, non-coding RNA; ‘PROT’, proteolysis or 26S proteasome function; ‘R/T/T’, replication, transcription, and translation; ‘ReDox’, reduction/oxidation and oxidoreductive stress; ‘SigT’ Signal Transduction; ‘UNK’, unknown functions.
