# Supplementary material for: Exon-Enriched Libraries Reveal Large Genic Differences Between Aedes aegypti from Senegal, West Africa, and Populations Outside Africa
Source: G3 (Bethesda). 2016 Dec 19;7(2):571–82. doi: 10.1534/g3.116.036053 (PMC5295602; doi:10.1534/g3.116.036053)
Supplement: Supplementary file 15 [file 571TableS4.pdf]

Table S4. Comparisons yielding upper 1% of FST values.

VectorBase	FST PK10 Mer	nSNPs	FST PK10 Kao	nSNPs	FST PK10 Thai	nSNPs	FST Kao Mer	nSNPs	FST Kao Thai	nSNPs	FST Mer Thai	nSNPs	Group	Annotation
AAEL001826	-	-	0.561	126	-	-	0.753	89	0.707	121	-	-	CSR	odorant-binding protein 56a, putative
AAEL015147	0.716	302	0.541	292	0.691	326	-	-	-	-	-	-	CSR	odorant receptor
AAEL017221	0.752	172	0.722	167	0.859	175	-	-	-	-	-	-	CSR	odorant receptor
AAEL009807	0.742	188	0.549	206	0.729	193	-	-	-	-	-	-	CYT/STR	cuticle protein, putative
AAEL010407	0.639	175	0.565	259	0.694	173	-	-	-	-	-	-	CYT/STR	claudin superfamily
AAEL011982	0.679	569	0.538	674	0.682	677	-	-	-	-	-	-	CYT/STR	cortactin
AAEL002781	0.752	99	0.686	109	0.737	104	-	-	-	-	-	-	DIV	galactokinase
AAEL003022	-	-	0.636	42	-	-	0.665	29	0.694	29	-	-	DIV	serine/threonine-protein kinase
AAEL004050	0.649	261	0.529	347	0.715	290	-	-	-	-	-	-	DIV	diphosphomevalonate decarboxylase
AAEL004240	0.658	234	0.545	263	0.699	289	-	-	-	-	-	-	DIV	gamma glutamyl transpeptidases
AAEL005117	-	-	-	-	0.803	98	-	-	0.917	79	0.863	73	DIV	5p-1878|hypo protein
AAEL009759	0.637	150	0.523	134	0.740	156	-	-	-	-	-	-	DIV	Mito. Distribution/morphology, apoptosis
AAEL010417	0.771	108	0.711	133	0.762	111	-	-	-	-	-	-	DIV	phosphotransferase
AAEL010477	0.703	105	0.675	137	0.758	9	-	-	-	-	-	-	DIV	Hotdog domain
AAEL011972	0.826	148	0.604	176	0.796	174	-	-	-	-	-	-	DIV	actin binding protein, putative
AAEL013152	0.657	575	0.555	641	0.723	595	-	-	-	-	-	-	DIV	Extensin
AAEL013157	0.692	352	0.665	399	0.755	389	-	-	-	-	-	-	DIV	Extensin
AAEL013330	0.640	89	0.543	98	0.685	94	-	-	-	-	-	-	DIV	alkaline phosphatase
AAEL013658	-	-	-	-	0.704	102	-	-	0.816	84	0.882	51	DIV	conserved hypo protein
AAEL013764	-	-	-	-	0.759	112	-	-	0.768	56	0.713	29	DIV	WD-repeat protein
AAEL015053	-	-	0.665	28	-	-	0.758	19	0.808	2	-	-	DIV	2-oxo-4-hydroxy-4-carboxy-5-ureidoimidazoline decarboxylase
AAEL015481	0.847	30	0.844	28	0.797	31	-	-	-	-	-	-	DIV	neuronal calcium sensor, putative
AAEL007336	0.889	40	-	-	-	-	0.929	31	-	-	0.876	28	PROT	ubiquitin-conjugating enzyme E2 q
AAEL007992	0.754	172	0.633	204	0.673	189	-	-	-	-	-	-	PROT	cysteine/serine peptidase

AAEL007946	0.701	367	-	-	-	-	0.690	340	-	-	0.883	352	ReDox	GSTE6
AAEL007948	-	-	-	-	0.666	138	-	-	0.690	136	0.897	166	ReDox	GSTE7
AAEL007951	-	-	-	-	0.727	315	-	-	0.787	291	0.922	375	ReDox	GSTE2
AAEL007962	0.699	266	-	-	-	-	0.759	231	-	-	0.901	264	ReDox	GSTE4
AAEL012491	0.634	214	0.639	159	0.792	224	-	-	-	-	-	-	ReDox	CYP6P12
AAEL014891	0.673	241	0.634	341	0.768	297	0.682	241	0.700	303	0.740	206	ReDox	CYP6P12
AAEL012638	-	-	0.650	24	-	-	0.849	18	0.769	18	-	-	R/T/T	nuclease HARBI1
AAEL013154	0.676	236	0.709	212	0.707	246	-	-	-	-	-	-	R/T/T	Zinc finger
AAEL013687	0.640	16	-	-	-	-	0.839	31	-	-	0.698	21	R/T/T	SAGA-associated factor 29
AAEL016997	-	-	-	-	0.831	105	-	-	0.885	97	0.550	74	SigT	GPCR Gastrin/Bombesin Family
AAEL017410	-	-	-	-	0.678	100	-	-	0.723	82	0.701	69	SigT	GPCR Gastrin/Bombesin Family
AAEL007950	-	-	-	-	0.674	409	-	-	0.681	293	0.696	268	TRP	Epidermal growth factor receptor substrate 15-like
AAEL014911	0.656	163	0.677	170	0.692	159	-	-	-	-	-	-	TRP	synaptic vesicle protein
AAEL000096	0.654	94	-	-	-	-	0.688	72	-	-	0.745	74	UNK	conserved hypo protein
AAEL001911	0.844	94	0.560	97	0.851	110	-	-	-	-	-	-	UNK	hypo protein
AAEL002905	0.745	175	0.783	192	0.686	204	-	-	-	-	-	-	UNK	conserved hypo protein
AAEL004525	0.653	146	0.632	168	0.720	163	-	-	-	-	-	-	UNK	hypo protein
AAEL007791	0.697	251	0.656	293	0.714	280	-	-	-	-	-	-	UNK	conserved hypo protein
AAEL013846	0.718	102	0.749	116	0.799	102	-	-	-	-	-	-	UNK	hypo protein
AAEL014972	-	-	0.504	19	-	-	0.792	14	0.724	9	-	-	UNK	hypo protein
AAEL015114	-	-	-	-	0.668	174	-	-	0.834	142	0.722	98	UNK	conserved hypo protein
AAEL015451	0.738	61	0.554	46	0.732	54	-	-	-	-	-	-	UNK	hypo protein
AAEL015509	-	-	-	-	0.669	28	-	-	0.745	15	0.582	14	UNK	conserved hypo protein
AAEL017937	0.796	15	0.669	14	0.816	9	-	-	-	-	-	-	UNK	hypo protein

* Functional classes: ‘CSR’, chemosensory reception; ‘CYT/STR’, cytoskeletal and structural; ‘DIV’, diverse functions; ‘PROT’, proteolysis or 26S proteasome function; ‘R/T/T’, replication, transcription, and translation; ‘ReDox’, reduction/oxidation and oxidoreductive stress; ‘SigT’, Signal Transduction; ‘TRP’, transport or signaling; ‘UNK’, unknown functions.
